# Sijunzi Decoction Reverses Metabolic Adaptation and Induces Ferroptosis in Cisplatin-Resistant Non–small Cell Lung Cancer: An Integrative Metabolomics–Pharmacology Analysis

**DOI:** 10.1007/s11596-026-00203-x

**Published:** 2026-05-13

**Authors:** Wen-jun Liu, Han-yu Dong, Chun-ying Liu, Chun Wang

**Affiliations:** 1 Key Laboratory of Ministry of Education for Traditional Chinese Medicine (TCM) Viscera-State Theory and Applications, Liaoning University of Traditional Chinese Medicine, Shenyang, 110847 China; 2 School of Basic Medical Sciences, Liaoning University of Traditional Chinese Medicine, Shenyang, 110847 China; 3 Teaching and Experimental Center, Liaoning University of Traditional Chinese Medicine, Shenyang, 110847 China

**Keywords:** Cisplatin resistance, Non-small cell lung cancer, Sijunzi decoction, Metabolic reprogramming, Adaptive response, Ferroptosis

## Abstract

**Objective:**

To investigate the mechanistic role of Sijunzi decoction (SJZD) in overcoming chemoresistance through the suppression of adaptive metabolic responses in non–small cell lung cancer (NSCLC).

**Methods:**

Chemical profiling of SJZD-derived components in systemic circulation was conducted using liquid chromatography–tandem mass spectrometry (LC‒MS/MS) in Sprague–Dawley rats. Multiomics integration and network pharmacology were employed to identify convergent targets shared by the bioactive constituents of SJZD and genes associated with cisplatin resistance. *In vitro* functional assessments using cisplatin-resistant human lung adenocarcinoma (A549/DDP) cells included the following: quantification of cell viability via Cell Counting Kit-8 (CCK-8) assays; evaluation of mitochondrial bioenergetics through targeted metabolomic profiling; and ultrastructural characterization of ferroptotic morphology via transmission electron microscopy (TEM). Cellular redox homeostasis was dynamically monitored using fluorescent probes, including a DCFH-DA probe for reactive oxygen species (ROS) and a C11-BODIPY^581/591^ probe for lipid peroxidation. siRNA-mediated gene silencing and immunohistochemical analysis were performed to elucidate the functional hierarchy of the p62/Keap1/nuclear factor erythroid 2-related factor 2 (Nrf2) antioxidant axis. Complementary *in vivo* validation was performed using BALB/c nude mice bearing A549/DDP xenografts, with longitudinal monitoring of tumor progression under SJZD treatment regimens.

**Results:**

Untargeted metabolomics of SJZD-medicated serum revealed 392 differentially abundant metabolites, with pathway enrichment revealing significant dysregulation of glutamine metabolism. Structural validation confirmed 55 bioactive components of SJZD in serum, including glycyrrhizin, ginsenoside Ro, liquiritigenin, and atractylenolide I. Integration of these components with disease targets yielded 355 overlapping genes associated with both SJZD activity and cisplatin-resistant NSCLC, with significant enrichment in oxidative stress response pathways. Experimental assays confirmed that SJZD induced ferroptosis in cisplatin-resistant A549/DDP cells, as evidenced by disrupted iron homeostasis, lipid peroxidation, and characteristic mitochondrial damage. These effects and subsequent cell death were specifically abrogated by the ferroptosis inhibitor ferrostatin-1 (Fer-1) but not by apoptosis inhibition, confirming that ferroptosis is the primary mechanism of cell death. Mechanistically, the inhibition of p62/Keap1/Nrf2 signaling was involved in the modulation of SJZD-induced ferroptosis both *in vitro* and *in vivo*.

**Conclusions:**

SJZD counteracts metabolic adaptation through ferroptosis mediated by the inhibition of p62/Keap1/Nrf2 in cisplatin-resistant NSCLC.

**Supplementary Information:**

The online version contains supplementary material available at 10.1007/s11596-026-00203-x.

## Introduction

Despite considerable therapeutic advancements, cancer remains the second leading cause of global mortality. Non–small cell lung cancer (NSCLC) accounts for 85% of lung cancer cases, and nearly one-third of patients have locally advanced disease [1]. Platinum-based chemotherapy is a standard treatment for locally advanced NSCLC, but chemoresistance substantially compromises clinical efficacy [2, 3]. Consequently, developing novel combination strategies to overcome chemoresistance is imperative.

The major goal of chemotherapy is to enable the powerful apoptosis of tumor cells predominantly through cytotoxic mechanisms targeting mitochondrial DNA (mtDNA) [2, 4]. Mitochondria are primarily responsible for meeting the metabolic demands of the tricarboxylic acid (TCA) cycle and oxidative phosphorylation (OXPHOS), and they also play a key role in regulating adaptive metabolic responses that help counteract cytotoxic stress. Paradoxically, this adaptive capacity can lead to chemotherapy resistance and treatment failure [5, 6]. Unlike adjacent normal tissues, tumors exhibit enhanced glycolysis and glutaminolysis, along with attenuated OXPHOS—metabolic adaptations that are critical for supporting tumor growth and proliferation [7]. Glutamine levels are significantly lower in the core regions of the tumor than in the periphery [6]. Obrist et al. reported that glutamine deprivation enhances cisplatin sensitivity in NSCLC [8]. Conversely, drug-resistant cells often respond adaptively, resulting in treatment failure [9]. Consistent with these findings, our prior work demonstrated that glutamine deprivation enhances cisplatin sensitivity in NSCLC. However, cisplatin-resistant NSCLC cells develop phenotypic resistance to cisplatin and exhibit an enhanced adaptive response during glutamine deprivation [10]. This interplay underscores the underlying complexity of treating drug-resistant cells.

Emerging evidence highlights that evasion of ferroptosis is a critical mechanism in lung cancer chemoresistance, suggesting that reactivating this process may offer a promising therapeutic strategy for overcoming drug resistance in NSCLC [11–13]. Mechanistically, ferroptosis is intrinsically associated with cellular energy metabolism [14–16]. Intriguingly, traditional Chinese medicine (TCM) and its bioactive constituents, which have long been utilized in oncology for their polypharmacological actions and favorable safety profiles [13], are being increasingly recognized as potent modulators of energy metabolism. Given that ferroptosis is intricately linked to metabolic pathways, this modulatory capacity positions TCM as a promising source of ferroptosis-inducing agents to counteract chemoresistance. Sijunzi decoction (SJZD), originating from the Formulary of the Peaceful Benevolent Dispensary (*Taiping Huimin Heji Jufang)*, is a classical prescription for invigorating spleen Qi, which comprises *Panax ginseng* C.A. Mey., *Atractylodes macrocephala* Koidz., *Wolfiporia extensa* (Peck) Ginns, and *Glycyrrhiza uralensis* Fisch. Ex DC. Pharmacology studies have demonstrated its efficacy in ameliorating chemotherapy-induced deterioration in quality of life. Consequently, SJZD is extensively employed as an adjunctive therapy to chemotherapeutic regimens in clinical cancer treatment [17–19]. However, the mechanistic role of SJZD in overcoming cisplatin resistance in NSCLC remains undefined. Here, we demonstrate that SJZD counteracts metabolic adaptation in cisplatin-resistant NSCLC by specifically inducing ferroptosis. Our findings establish that combining platinum-based chemotherapy with targeted metabolic modulation, particularly ferroptosis induction, represents a more effective therapeutic strategy. These insights underscore the potential of SJZD as an adjunctive agent to improve therapeutic outcomes in patients with cisplatin-resistant NSCLC.

## Materials and Methods

### Reagents and Antibodies

*Panax ginseng* C.A. Mey. (#2020050), *Atractylodes macrocephala* Koidz. (#2011240), *Wolfiporia extensa* (Peck) Ginns (#2107160), and *Glycyrrhiza uralensis* Fisch. ex DC. (#2101001) were purchased from Beijing Tong Ren Tang (China). The components of the herbal formulation are summarized in Table 1. The predominant reagents used were L-glutamine (#C0212; Beyotime Institute of Biotechnology, China) and cisplatin (P4394; Sigma‒Aldrich, Germany). Matrigel basement membrane matrix (#356234) was obtained from Corning Labware Products, Inc. (Corning, USA), and pentobarbital sodium from Merck KgGA (Germany) was used. A short interfering ribonucleic acid (siRNA) kit targeting Keap1 (#stB0009190A) was purchased from RiboBio Technology Co., Ltd. (China). Golden Trans DR Reagent (#PE-401—3001) was obtained from Golden Trans Technology Co., Ltd. (China). A Cell Counting Kit-8 (CCK-8; #C0037), an adenosine triphosphate (ATP) Assay Kit (#S0026), a mitochondrial membrane potential (MMP) Assay Kit with tetraethylbenzimidazolylcarbocyanine iodide (JC-1; #C2006), a ROS Assay Kit (#S0033S), a terminal deoxynucleotidyl transferase-mediated nick-end labeling (TUNEL) Cell Apoptosis Assay Kit (#C1086), and Ki-67 antibody were obtained from Beyotime. We purchased a reduced glutathione (GSH) assay kit (#A006) from Nanjing Jiancheng Bioengineering Institute Co., Ltd. (China), an iron colorimetric assay kit (#E-BC-K139-S), and a lactic acid (LLA) colorimetric assay kit (#E-BC-K004-M) from Elabscience (USA). C11-BODIPY^581/591^ (#D3861) and a MitoTracker probe (#M7512) were purchased from Invitrogen (Thermo Fisher Scientific, USA). FerroOrange probe (#F374) and malondialdehyde (MDA) assay kits (#M496) were purchased from Dojindo Laboratories (Japan). We purchased primary antibodies against ferritin-heavy chain (FTH; #4393), divalent metal transporter 1 (DMT1; #15083), glucose transporter 1 (GLUT1; #73015S), pyruvate dehydrogenase (PDH, #3205), lactate dehydrogenase A (LDHA (#2012), hexokinase 1 (HK1; #2804), HK2 (#2106), pyruvate kinase muscle isozyme M2 (PKM2; #3198), poly(adenosine diphosphate [ADP]–ribose) polymerase (PARP; #9542), and a p62/KEAP1/NRF2 Pathway Antibody Sampler Kit (#48768) from Cell Signaling Technology (CST; USA). Antibodies against succinate dehydrogenase complex, subunit A, flavoprotein variant (SDHA; #14865—1-AP), SDHB (#10620—1-AP), cysteine–aspartic acid–specific protease/proteinase 3 (Caspase-3; #19677—1-AP), B-cell lymphoma 2 (Bcl-2; #12789—1-AP), Bcl-2-like protein 4 (Bax; #50599—2-Ig), ferroportin (FPN; #26601—1-AP), xCT (#26864—1-AP), GPX4 (#67763—1-AP), ACSL4 (#22401—1-AP), hypoxia inducible factor 1, alpha subunit (HIF-1α; #20960—1-AP), and β-actin (#66009—1-Ig) were obtained from Proteintech, China. We procured the translocase of outer mitochondrial membrane 40 homolog (TOMM20; #ab186735), cleaved caspase-3 (#ab32042), light chain 3B (LC3B; #48394), and total OXPHOS complex (#110413) from Abcam (Cambridge, UK). Transferrin receptor protein 1 (TfR-1; #13—6800) was obtained from Invitrogen (Carlsbad, CA, USA). ECL signals were detected following Western blotting analysis using a chemiluminescent substrate (#1805001; Tanon Science & Technology Co., Ltd., China).

**Table 1:** Composition of the Sijunzi decoction

Botanical name	English herbal name	Chinese name	Medicinal parts	Dosage
*Panax ginseng* C.A.Mey	Ginseng	Ren Shen	Radix et Rhizoma	9 g
*Atractylodes macrocephala* Koidz	White Atractylodes Rhizome	Bai Zhu	Rhizoma	9 g
*Wolfiporia extensa* (Peck) Ginns	Poria Sclerotium	Fu Ling	Sclerotium	9 g
*Glycyrrhiza uralensis* Fisch. ex DC	Honey-fried Licorice	Gan Cao	Radix et Rhizoma Praeparata	6 g

### Cell Culture, Glutamine Deprivation, and Inhibitor Treatment

Both A549 and A549/DDP (cisplatin-resistant A549 lung adenocarcinoma cells) cell lines were purchased from Beijing Dingguo Changsheng Biotechnology Co., Ltd. (China) and were used within three months of thawing. The cells were screened for *Mycoplasma* using a One-step Quickcolor *Mycoplasma* Detection Kit (#MD001; Shanghai YISE Economic & Trade Co., Ltd., China). Both cell lines were maintained in glutamine-free Dulbecco’s modified Eagle’s medium/nutrient mixture F-12 (DMEM/F12; #PM150313; ProCell Therapies [Clarion Medical Technologies Canada]) supplemented with L-glutamine (2 mM), 10% fetal bovine serum (FBS; #FS301—02; TransGen Biotech, Inc., China), 100 U/mL penicillin, and 100 mg/mL streptomycin solution (#SV30010; HyClone [GE Healthcare, USA]) at 37 °C in a 5% CO_2_ incubator. Drug-resistant A549/DDP cells were maintained in complete medium supplemented with 2 μg/mL cisplatin without penicillin–streptomycin antibiotics and were withdrawn 2 weeks before the experiment, as previously reported [15].

For SJZD-mediated serum treatment, we replaced the culture medium with complete medium containing 10% SJZD-medicated serum and cisplatin instead of 10% FBS and cisplatin in glutamine-free medium for 48 h.

### Cell Viability and Calcein/Propidium Iodide (PI) Double Staining Assay

We plated cells in 96-well plates (5 × 10^3^/well) and confirmed their adhesion before 24/48 h of treatment. Cell viability was quantified using the CCK-8 assay: 10 μL of CCK-8 reagent was added to each well, followed by incubation at 37°C for 1 h. Absorbance was then measured at 450 nm using a Tecan Infinite 200 PRO microplate reader (Switzerland). Cells (2 × 10^4^ cells/mL) were also seeded in a 24-well plate, after which a calcein AM/PI staining assay was performed. After incubation (30 min at 37 °C), images were acquired using a Bio-Rad ZOE Imager (Hercules, USA).

### Transmission Electron Microscopy (TEM)

For TEM, the samples were fixed in 2.5% glutaraldehyde (30 min, room temperature). Following dehydration through an ethanol gradient, the samples were embedded in EPON resin (Hexion Inc., USA). Ultrathin sections were doubly stained with 2% uranyl acetate and lead citrate before TEM imaging (Hitachi JEM-1200EX, Japan). Quantitative analysis was conducted by calculating the percentage of mitochondria exhibiting characteristic ferroptotic morphology (e.g., shrunken mitochondria with increased membrane density and reduced cristae) in each group.

### Hematoxylin/Eosin Staining, Immunohistochemical, TUNEL, and Immunofluorescence Staining

Paraffin-embedded sections were dewaxed, rehydrated, and rinsed with phosphate-buffered saline (PBS), followed by hematoxylin and eosin (H&E) staining (#C0105S; Beyotime). To quench endogenous peroxidase activity, the sections were treated with 3% H_2_O_2_, DNase-free proteinase K (#ST532, Beyotime) (37°C, 25 min) or QuickBlock Blocking Buffer (#P0260; Beyotime). After blocking, the sections were incubated overnight at 4°C with primary antibodies against Ki-67, xCT, Keap1, and Nrf2 (1:200) or with TUNEL detection solution at 37°C for 1 h. Subsequently, the sections were incubated with the appropriate secondary antibody (37°C, 1 h), followed by chromogenic development using DAB (#SV0004; Boster Biological Tech, China) or 4′,6-diamidino-2-phenylindole (DAPI; #P0131; Beyotime). Finally, the slides were imaged using either brightfield microscopy or an Olympus FV10i confocal microscope (Olympus Corp., Japan).

### siRNA Transfection

Cells were seeded overnight in 96-well plates (5 × 10^3^ cells/well). Transfections were performed 24 h prior to treatment with SJZD-medicated serum, using Golden Trans DR Reagent with either *Keap1*-targeting siRNA (sequence: GCTACGATGTGGAAACAGA; ID: stB0009190A) or nontargeting control siRNA (RiboBio). Following 48 h of treatment, cell viability was quantified using CCK-8 assay. Keap1 knockdown efficiency was validated by immunoblotting following the manufacturer's protocols.

### Adenosine Triphosphate Levels and Iron Colorimetric Assay

Cells were harvested by scraping and then were lysed on ice in ATP-specific buffer or PBS for the iron colorimetric assay. The cell lysis solution was then centrifuged (12,000 g, 4°C, 5 min), and the supernatant (20 μL) was mixed with 100 μL of working solution in black microwell plates or 600 μL of chromogenic reagent. Finally, the luminescence (SpectraMax i3X, Molecular Devices, USA) or OD at 520 nm (Tecan microplate reader) was determined.

### Mitochondrial-Membrane Potential, FerroOrange, Lipid Peroxidation, and Reactive Oxygen Species Assays

Cells were seeded in 24-well plates (2 × 10^4^ cells/mL), precultured in glutamine-free medium for 6 h, and treated for 48 h. After being washed, the cells were stained with FerroOrange (1 μM) or C11-BODIPY^581/591^ (5 μM) or DCFH-DA (1 μM), Hoechst 33,342 (10 μg/mL), and either MitoTracker (1 μM) or JC-1 (1:1000); fluorescence was quantified (SpectraMax i3X) or imaged (Bio-Rad ZOE Fluorescent Cell Imager).

### Western Blotting Analysis

The samples were subsequently washed twice with cold PBS and lysed in RIPA buffer (#P0013B; Beyotime) supplemented with protease inhibitors (200 mM AEBSF, 30 µM aprotinin, 13 mM bestatin, 1.4 mM E64, and 1 mM leupeptin). After protein concentrations were determined by BCA assay, equal amounts (25 µg) underwent SDS‒PAGE and electrotransfer to PVDF membranes (0.25 µm; Bio-Rad). The membranes were then blocked with 5% BSA/TBST (1 h, RT), incubated with primary antibodies (1:1000, 4°C, overnight) and the appropriate secondary antibodies (1:5000, RT, 1 h), and then detected using ECL reagent. Blots were visualized (Tanon 5200 imager), quantified with AlphaView software (v3.4.0.0; ProteinSimple) and normalized to β-actin.

### Animal Studies

#### Ethics Statement

In compliance with China’s Institutional Animal Care Guidelines, this study received ethical approval (No. 21000042021107) from Liaoning University of TCM’s Animal Care & Welfare Committee (China). Animal suffering was minimized through standardized analgesic protocols and humane endpoint criteria.

#### Preparation of SJZD-Medicated Serum

To prepare the SJZD extract, the raw materials of *P. ginseng* (9 g)*, A. macrocephala* (9 g)*, P. cocos* (9 g)*,* and *G. uralensis* (6 g) were weighed and soaked in 1500 mL of distilled water for 30 min. The mixture was then subjected to primary reflux extraction at 100°C for 45 min. The filtrate was collected, and the residues underwent secondary extraction with 1200 mL distilled water at 100°C for 30 min. Both filtrates were then combined and concentrated to a final volume of 100 mL, yielding a stock solution of 4.25 g/mL. This stock solution was subsequently diluted with 0.9% normal saline to obtain working concentrations of 2.125, 1.0625, and 0.2125 g/mL. For subsequent animal studies, the dosage of SJZD for Sprague‒Dawley rats was calculated using the human equivalent dose per body surface area [20]. According to the *Pharmacopoeia of the People’s Republic of China* (2020 Edition), the recommended clinical dosage of SJZD for humans is 33 g/60 kg/day. By applying the standard conversion factor (approximately 6.3 for rats on the basis of body surface area [21]), the equivalent dose for rats was calculated to be 3.40 g/kg.

Based on the calculated dosages, the *in vivo* study was conducted as follows. Twenty-four male rats (average body weight [BW]: 250 g) were acclimated for one week under standard conditions and subsequently randomized into four groups: normal saline, high-dose SJZD (34 g/kg, 2.125 g/mL, 10 ×), medium-dose SJZD (17 g/kg, 1.0625 g/mL, 5 ×), and low-dose SJZD (3.40 g/kg, 0.2125 g/mL, 1 ×). All of the treatment groups received daily intragastric (i.g.) administration of 4.0 mL of the respective treatments (SJZD or 0.9% normal saline) once daily for seven consecutive days. One hour after the final gavage, the animals were anesthetized, and the abdominal cavity was exposed to collect whole blood from the abdominal aorta. Blood samples were allowed to clot and subsequently centrifuged at 2500 r/min for 10 min. Serum was collected, heat-inactivated at 56 °C in a water bath for 30 min, filtered through a 0.22-µm pore-size membrane filter (Bio-Rad Laboratories), and stored at −20 °C for subsequent analysis. A 10% serum concentration was utilized for *in vitro* analysis.

#### Identification of SJZD Absorbed into Rat Blood

Briefly, SJZD-treated serum was mixed with 80 μL of hydrochloric acid solution (2 mol/L). After vortexing and centrifuging, the supernatant was collected under nitrogen. The resulting residue was reconstituted in 100 μL of extraction solvent (MeOH:ACN:H_2_O, 2:2:1, v/v). LC‒MS/MS analysis was performed on a Vanquish UHPLC system (Thermo Fisher) coupled to an Orbitrap Exploris 120 mass spectrometer using a Phenomenex Kinetex C18 column (2.1 × 100 mm, 2.6 μm). Chromatographic separation was as follows: mobile phase A: 0.01% acetic acid in water; mobile phase B: IPA:ACN (1:1, v/v). The autosampler temperature was 4 °C, and the injection volume was 2 μL.

#### SJZD Treatment

We obtained 32 BALB/c nude mice (6 weeks; M/F = 1:1) from HFK Bioscience (China) with 3 days of IVC acclimatization (7.6-L cages; Shinva, China) preceding the studies. All 32 of the mice were subcutaneously inoculated in the upper left flank with 2 × 10^6^ A549/DDP cells mixed in glutamine-free DMEM/F12 and Matrigel basement membrane matrix at a 1:1 ratio. Each nude mouse was successfully inoculated, and when the length of the tumors reached approximately 5 mm, as determined with a caliper, the mice were randomized into control, cisplatin (CDDP), SJZD, and CDDP + SJZD combination treatment groups (n = 8 each) according to our previous work [15]. To minimize animal suffering, cleaning practices were regularly monitored to ensure effective hygiene and sterile sanitation. To avoid unnecessary harm, PBS, CDDP, and SJZD were injected gently daily via intraperitoneal (i.p.) injection or intragastric (i.g.) administration on a vertical laminar-flow clean workbench (BSE-CC-A 1000; Shinva).

Consistent with the preparation for the SJZD-medicated serum study, the *in vivo* dosage for mice was calculated on the basis of the clinical equivalent dose. In accordance with the human dose of 33 g/60 kg/day and using a standard conversion factor of 9.1 for mice on the basis of body surface area [21], the equivalent dose for mice was determined to be 5.005 g/kg. For the *in vivo* efficacy study, a high dose, equivalent to 10 times the clinical dose (50 g/kg), was administered. To achieve the required dosage for administration, the SJZD decoction was concentrated to 2.5 g/mL for gavage. The mice in the treatment groups received daily intragastric (i.g.) administration of 400 μL of SJZD or 0.9% normal saline for three consecutive weeks. Additionally, 50 mg/kg CDDP was administered intraperitoneally (i.p.) once per week over the same three-week period. At the end of the treatment period, the mice were euthanized with 1% pentobarbital sodium (50 mg/kg, i.p.), and the tumors were surgically resected and weighed.

### Network Pharmacology

The active components of *P. ginseng, A. macrocephala, P. cocos, and G. uralensis* were systematically retrieved from the TCMSP database (https://old.tcmsp-e.com/tcmsp.php). Initial screening was performed using oral bioavailability (OB) ≥ 30% and drug likeness (DL) ≥ 0.1. These filtered compounds were then combined with bioavailable prototype components of SJZD. Chemical structure data (SMILES format) of the integrated compounds were acquired from PubChem (https://pubchem.ncbi.nlm.nih.gov/) and subsequently processed through SwissTargetPrediction (http://www.swisstargetprediction.ch/), with species restricted to humans (*Homo sapiens*). Targets predicted with a probability > 0 were retained and cross-validated against RNA sequencing differential expression profiles [22] using Sangerbox 3.0 (http://sangerbox.com/home.html) to obtain overlapping genes. The shared targets underwent comprehensive functional annotation through Metascape (http://Metascape.org/gp/index.html), including Gene Ontology (GO) and KEGG pathway analyses. Visualization outputs were generated using Sangerbox 3.0. Protein–protein interaction (PPI) networks were constructed via the STRING database (https://cn.string-db.org/) (minimum required interaction score = 0.900, eliminating disconnected nodes) and refined in Cytoscape. The core network was constructed using CytoNCA, and six centrality metrics were evaluated: betweenness centrality, closeness centrality, degree centrality, eigenvector centrality, local average connectivity, and network centrality. Targets scoring below the median across all parameters were iteratively removed. Finally, an integrative pharmacological network was constructed in Cytoscape to map multilayered relationships among herbs, components, target proteins, pathways, and diseases.

### Statistical Analysis

The data are expressed as mean ± standard deviation (SD). Statistical comparisons among multiple groups were performed using one-way ANOVA, followed by Tukey’s HSD post hoc test for multiple comparisons. Statistical significance was set at *P* < 0.05. Statistical analyses were performed using SPSS (version 19.0; IBM Corp., USA) and GraphPad Prism (version 7.0; GraphPad Software, Inc., USA).

## Results

### Quality Control of SJZD

**Fig. 1:**
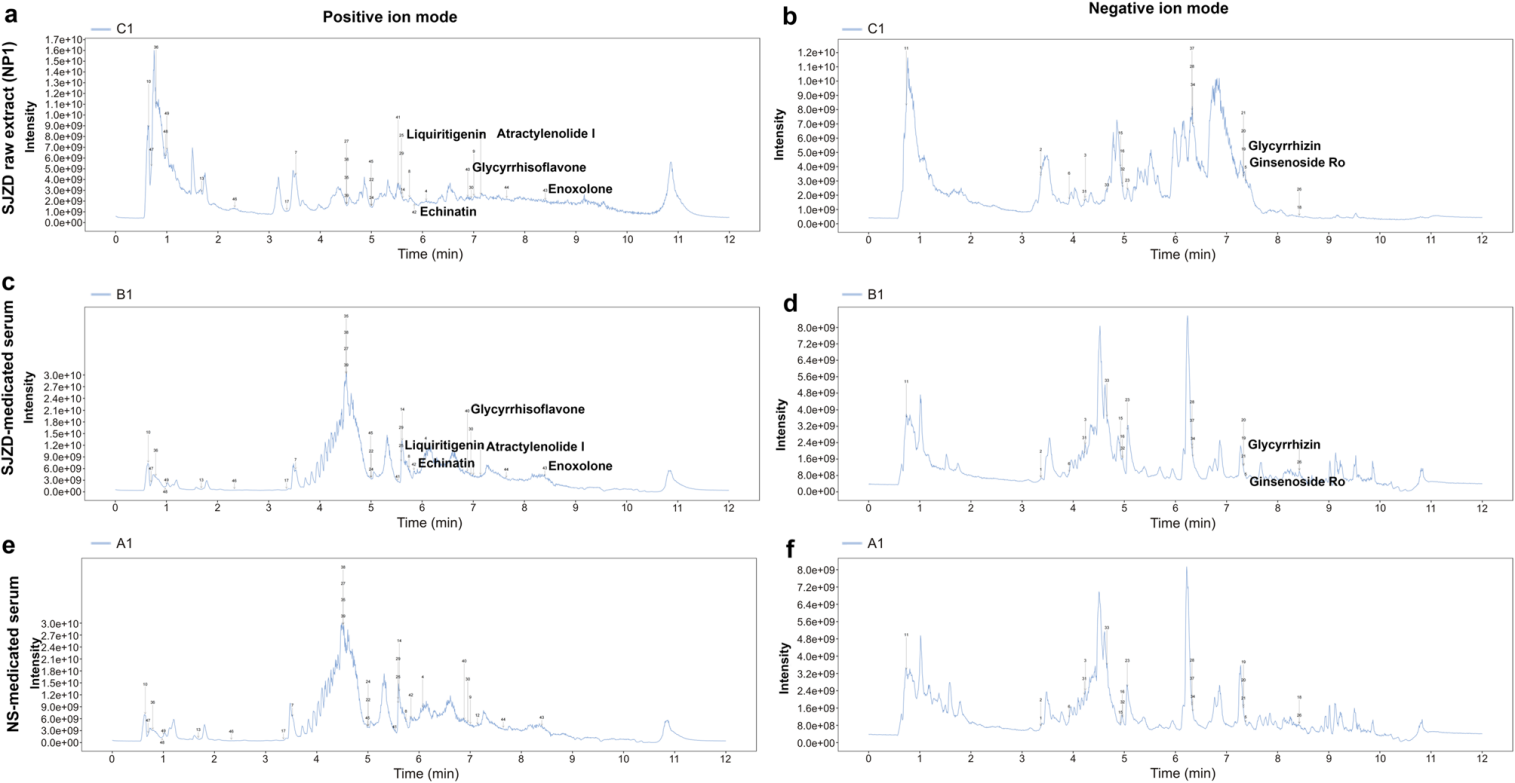
Total ion current chromatograms (TICs) of SJZD-medicated serum. Representative LC‒MS/MS TICs are presented for SJZD raw extract (**a, b**), SJZD-medicated serum (**c, d**), and NS-medicated serum (**e, f**). Panels (**a, c, e**) were acquired in positive ion mode; panels (**b, d, f**) were acquired in negative ion mode

LC‒MS/MS-based metabolomic profiling was systematically conducted to elucidate the predominant phytochemical constituents in SJZD raw extract (NP1), and we identified the bioactive components absorbed into systemic circulation through serum pharmacochemistry analysis, with SJZD-exposed rat plasma hereafter designated as SJZD-medicated serum. The characteristic chromatographic profiles and representative total ion chromatograms (TICs) in positive ion mode (detecting ginsenoside B, atractylenolide I, liquiritigenin, enoxolone, and glycyrrhisoflavone) and negative ion mode (identifying glycyrrhizin and ginsenoside Ro) were structurally validated in SJZD-medicated serum through reference standard alignment (Fig. 1a–f).

### Integrative Untargeted Metabolomics Identifies Dysregulated Glutamine Metabolism as a Key Signature in SJZD-Medicated Serum

**Fig. 2:**
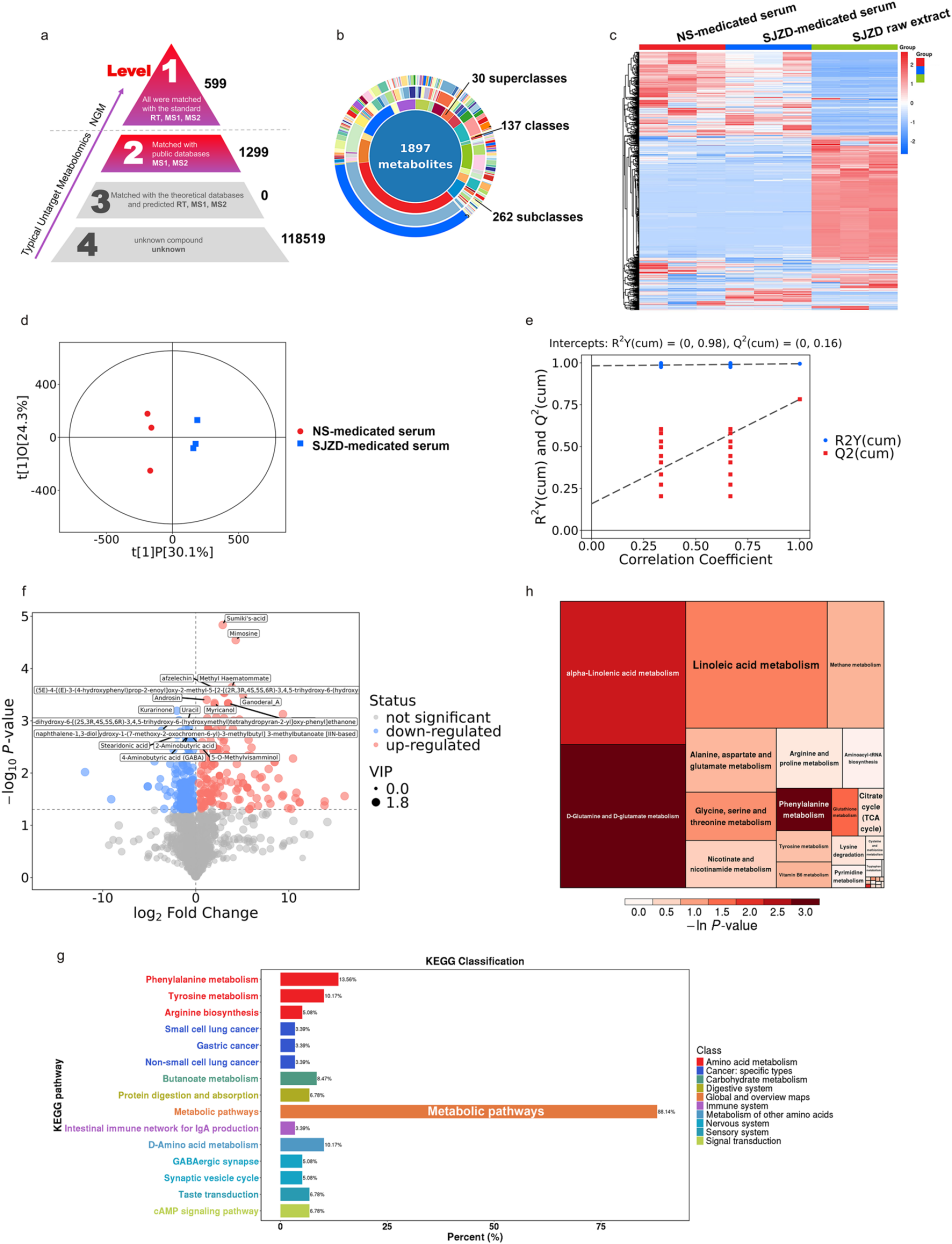
Multidimensional metabolomic profiling reveals distinct metabolic signatures and key pathways in SJZD-treated serum. **a** Funnel plot depicting the distribution and selection of metabolic features across SJZD-medicated serum, SJZD raw extract, and NS-medicated serum. **b** Donut plot of metabolite chemical classes in the three groups. **c** Heatmap with hierarchical clustering demonstrating clear segregation of the three groups on the basis of their metabolic signatures. **d** OPLS-DA score plots illustrating differences between the SJZD-medicated serum and NS-medicated serum groups. **e** Replacement test analysis of the SJZD-medicated serum group compared with the NS serum group. **f** Differentially abundant metabolites identified by nontargeted metabolomics, as shown by the volcano plot with VIP > 1 and *P* < 0.05 in both modes. **g** Heatmap of hierarchical cluster analysis of differentially abundant metabolites in both modes. **h** Tree map plot of pathways for the SJZD-medicated serum group vs. the NS-medicated serum group. Each square represents a metabolic pathway. The size of the square reflects the importance of the pathway in the topological analysis, while the color indicates the significance level of the enrichment, with deeper colors indicating more significant enrichment

LC‒MS-based untargeted metabolomics profiling was implemented across three experimental groups: NP1 (n = 3), SJZD-medicated serum (Sample 1, n = 3), and normal saline control serum (Blank, n = 3). Following rigorous data preprocessing, 120,396 metabolic features were retained, with 1897 putatively annotated metabolites (Fig. 2a–b). Hierarchical clustering analysis revealed distinct phylogenetic clustering patterns (Fig. 2c), demonstrating significant intergroup differential metabolic signatures (VIP > 1.0, *P* < 0.05). Orthogonal projections to latent structures-discriminant analysis (OPLS-DA) modeling revealed distinct metabolic distinctions between the SJZD-medicated serum group and the NS-control group, with all of the sample trajectories residing within Hotelling’s T2 95% confidence ellipse (Fig. 2d). To validate the model’s fidelity, 100-iteration permutation testing demonstrated that (1) the original model parameters (R^2^Y = 0.995, Q^2^ = 0.783) approached the theoretical maxima, confirming robust explanatory power (R^2^Y) and predictive capacity (Q^2^); (2) the randomized models exhibited significantly degraded performance (R^2^Y = 0.98, Q^2^ = 0.16, *P* < 0.001 via the Wilcoxon signed-rank test), confirming protection against overfitting (Fig. 2e). Multivariate feature selection revealed 392 differentially abundant metabolites (VIP > 1.0, FDR-adjusted *P* < 0.05), comprising 183 upregulated and 209 downregulated metabolites (Fig. 2f). The volcano plot highlights top-decile regulated metabolites across both ionization modes, with structural annotations verified through LC/MS spectral matching. KEGG pathway annotation revealed that 88.14% (n = 347/392) of the differentially abundant metabolites were mapped to metabolic pathways (Fig. 2g). Through integrated pathway impact analysis (*P* < 0.05, FDR < 0.1) and topological centrality evaluation (betweenness centrality > 0.8), three key dysregulated pathways emerged: D-glutamine and D-glutamate metabolism, alpha-linolenic acid metabolism, and phenylalanine metabolism (Fig. 2h).

### Integrated Network Pharmacology and Transcriptomic Analysis Reveal Oxidative Stress as a Core Mechanism Underlying the Therapeutic Effects of SJZD in Cisplatin-Resistant NSCLC

**Fig. 3:**
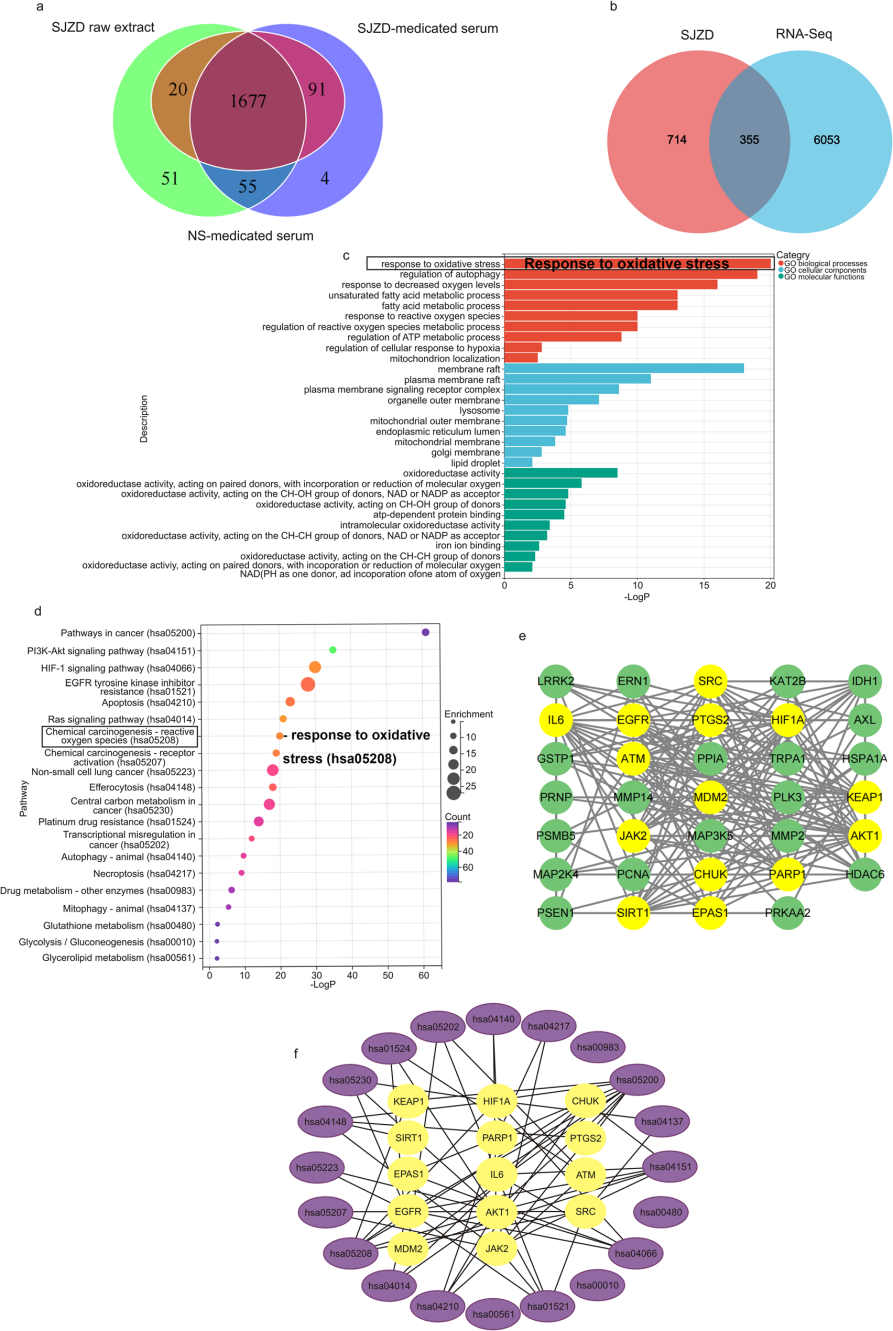
Prediction and analysis of interactions between SJZD metabolites and cisplatin-resistant NSCLC-related targets. **a** Fifty-five blood-entry prototype components identified by serum pharmacochemical analysis are shown in a Venn diagram. **b** Intersection analysis of SJZD components and differentially expressed genes in CDDP-resistant NSCLC based on our previous RNA-Seq analysis revealed enrichment, as shown by a Venn diagram. **c** A histogram of the results of the GO analysis of the target proteins; the top 10 enriched terms for biological process (BP), cellular component (CC), and molecular function (MF) are displayed. **d** KEGG pathway annotation (top 20 enriched pathways); the Y-axis label represents the signaling pathway, and the X-axis label represents the gene ratio. Gradual color changes represent probability changes. **e** Genes enriched in the “response to oxidative stress” pathway according to the results of the GO analysis were selected to construct a focused PPI subnetwork. **f** Network pharmacological map illustrating the relationships among hub targets and enriched KEGG pathways

Following comprehensive metabolomic characterization of SJZD raw extract, SJZD-medicated serum, and NS-medicated serum, integrated qualitative‒quantitative approaches revealed 55 bioavailable prototype phytochemicals that exhibited systemic absorption (Fig. 3a and Table S1). In parallel, 124 components were systematically retrieved from the TCMSP database. Integration of the metabolically active constituents of SJZD with TCMSP-derived compounds yielded 1069 potential therapeutic targets, and transcriptomic analysis of cisplatin-resistant A549/DDP cells revealed 6408 genes associated with drug resistance (Table S2). Intersection analysis revealed 355 overlapping genes potentially involved in both SJZD activity and cisplatin-resistant NSCLC pathogenesis (Fig. 3b).

GO enrichment analysis of these shared targets revealed significant associations with the response to oxidative stress in terms of biological processes (Fig. 3c). Notably, the enriched KEGG pathways included reactive oxygen species metabolism (Fig. 3d), suggesting that the therapeutic mechanism of SJZD involves the modulation of intracellular redox homeostasis. Consequently, we initially constructed a primary PPI network containing 269 targets interconnected by 739 edges (Fig. S1). Thirty-four genes enriched in the response to oxidative stress pathway were subsequently screened through GO analysis. Among them, 14 hub targets were selected, and a simplified network using hub targets/pathways was constructed, with the 14 core targets at the center and the enriched KEGG pathways as the outer circle (Fig. 3e–f). Collectively, these results strongly demonstrated that SJZD may induce oxidative stress in cisplatin-resistant NSCLC under glutamine deprivation conditions.

### SJZD Induces Oxidative Stress in Cisplatin-Resistant NSCLC Under Glutamine Deprivation

**Fig. 4:**
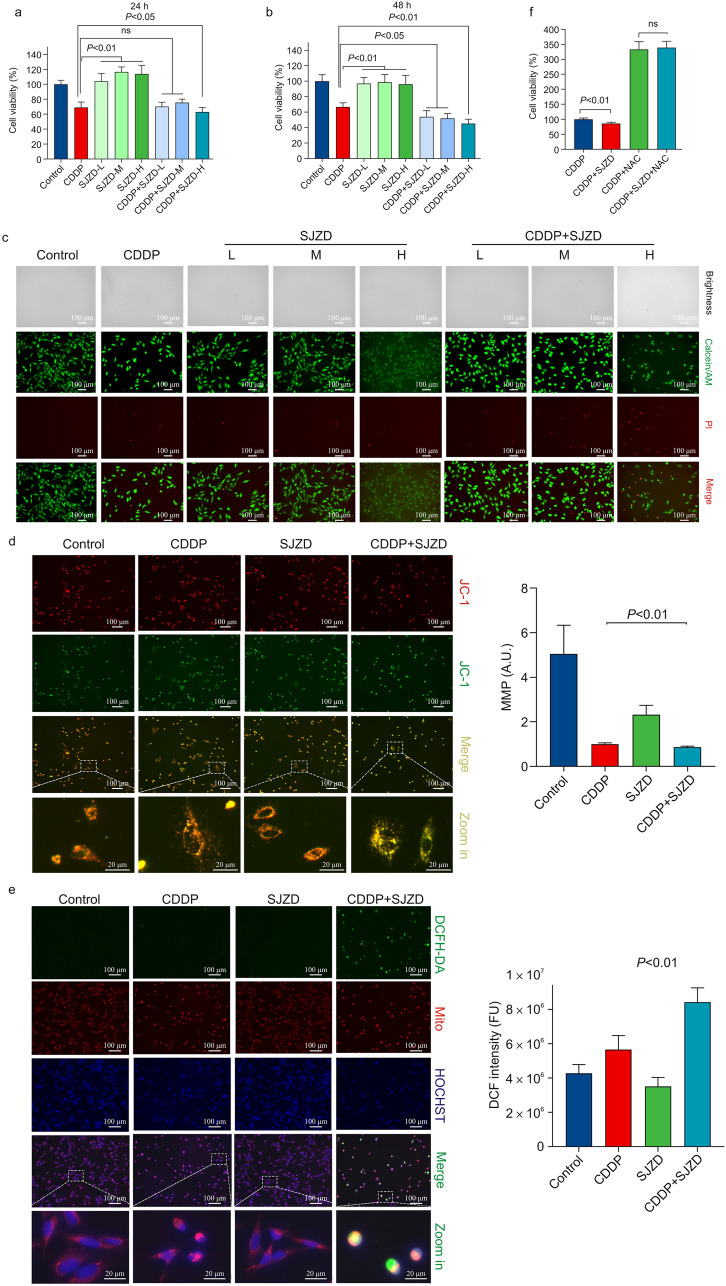
Cell viability and calcein/propidium iodide staining in glutamine-deprived A549/DDP cells were assessed following treatment with different concentrations of the SJZD combination treatment. Effects of different concentrations of SJZD-medicated serum (3.4 g/kg, 17 g/kg, 34 g/kg) + CDDP on cell viability in glutamine-deprived A549/DDP cells are shown at 24 h (**a**) and 48 h (**b**) (n = 3/group). **c** Glutamine-deprived A549/DDP cells treated with different concentrations of SJZD-medicated serum (3.4 g/kg, 17 g/kg, 34 g/kg) + CDDP were stained with calcein/propidium iodide (PI) for 48 h (n = 3/group). **d** Effect of SJZD combination (34 g/kg) on the MMP in glutamine-deprived A549/DDP cells was visualized by fluorescence microscopy and quantified using a fluorescence microplate and JC-1 staining (n = 3/group). **e** Effect of SJZD combination (34 g/kg) on mitochondrial ROS production in glutamine-deprived A549/DDP cells was visualized by fluorescence microscopy and quantified using DCFH-DA, MitoTracker, and Hoechst 33342 staining and a fluorescence microplate (n = 3/group). **f** Effect of NAC (5 mM; 48 h) on cell viability in glutamine-deprived A549/DDP cells after SJZD combination treatment (34 g/kg), as shown by CCK-8 assay (n = 3/group). The data represent mean ± SD

Functional validation commenced with CCK-8 cytotoxicity assays, which revealed that SJZD-medicated serum synergistically enhanced CDDP efficacy in a time-concentration reciprocity model. Under glutamine-deprived conditions mimicking tumor microenvironment stress, compared with CDDP monotherapy, the SJZD-CDDP combination resulted in a 32.0% reduction in viability at 48 h (*P* < 0.01), indicating its pharmacodynamic superiority (Fig. 4a–b). Complementary calcein-AM/PI dual fluorescence imaging further revealed the therapeutic synergy (Fig. 4c). Assessment of mitochondrial integrity via JC-1 fluorescence revealed marked depolarization in combination-treated cells, as evidenced by a 13.74% increase in the green/red fluorescence ratio compared with that in cells treated with CDDP alone (*P* < 0.01; Fig. 4d). Subsequent oxidative stress profiling demonstrated a 48.81% increase in DCFH-DA (*P* < 0.01; Fig. 4e). Oxidative stress was further confirmed by the complete reversal of the combined effects of CDDP monotherapy and SJZD-CDDP with 5 mM N-acetyl-L-cysteine (NAC) pretreatment (Fig. 4f). Together, these results strongly demonstrated that SJZD induces oxidative stress in cisplatin-resistant NSCLC cells under glutamine deprivation conditions.

### SJZD Attenuates TCA Cycle Metabolites in Cisplatin-Resistant NSCLC Under Glutamine Deprivation

**Fig. 5:**
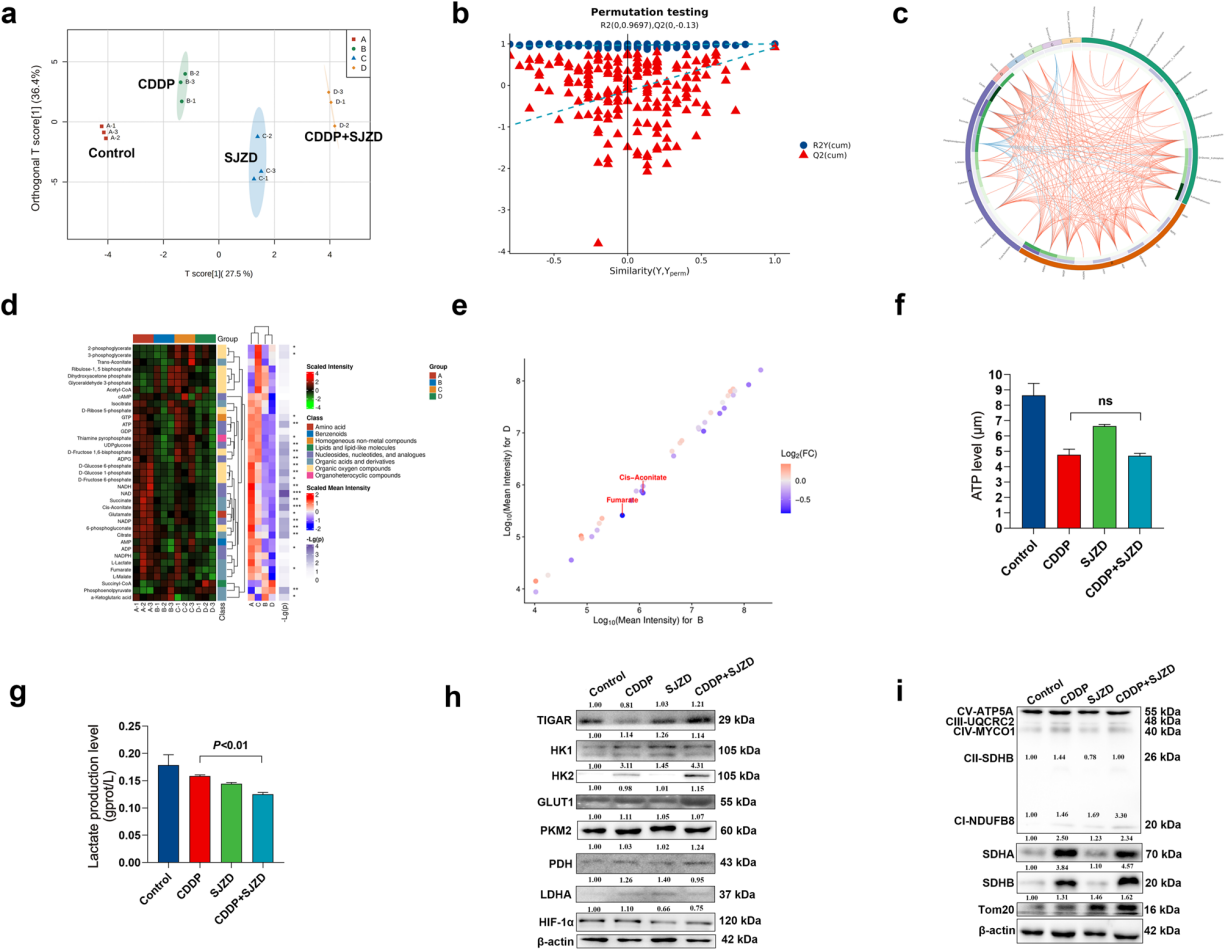
Effects of combined treatment with SJZD and CDDP on the activity of TCA cycle metabolites and the glycolytic pathway in glutamine-deprived A549/DDP cells. **a** OPLS-DA score, **b** permutation test, **c** Circos map, and **d** heatmap of the four groups of glutamine-deprived A549/DDP cells. **e** Metabolites with significant differences were screened out by one-way ANOVA. A–D represent the control, CDDP, SJZD and SJZD + CDDP groups, as shown in a scatter diagram (n = 3/group). A colorimetric method was used to investigate the effects of SJZD + CDDP on ATP levels (**f**) and lactate production (**g**) in glutamine-deprived A549/DDP cells (n = 3/group). Effects of SJZD in combination treatment (34 g/kg) on the expression of proteins related to aerobic glycolysis (**h**) and the mitochondrial respiratory chain (**i**) in glutamine-deprived A549/DDP cells were detected by WB (n = 3/group). The data represent mean ± SD

Guided by serum pharmacochemistry findings indicating D-glutamine and D-glutamate metabolism modulation (Fig. 2h), quantitative targeted metabolomics profiling revealed profound TCA cycle remodeling in combination-treated cells compared with that in cells treated with CDDP alone: the levels of cis-aconitate (−39.97%) and fumarate (−45.05%) were significantly decreased (all *P* < 0.05), despite unchanged ADP/ATP ratios (*P* > 0.05; Fig. 5a–e; Tables S3, S4) and preserved ATP homeostasis (*P* > 0.05; Fig. 5f). This metabolic signature was corroborated by a decrease in glycolytic flux (21.03% reduction in extracellular lactate accumulation; *P* < 0.01; Fig. 5g), upregulation of glucose-fuelled mitochondrial respiration (increased expression of HK2, TIGAR, GLUT1, PDH, NDUFB8, and SDHB; Fig. 5h–i), and downregulation of aerobic glycolysis (suppression of HIF-1α and LDHA; Fig. 5h), demonstrating the unique ability of SJZD to attenuate the activity of TCA cycle metabolites while promoting glucose-fuelled mitochondrial respiration.

### SJZD Induces Iron Metabolism Imbalance and Ferroptosis in Cisplatin-Resistant NSCLC Under Glutamine Deprivation

**Fig. 6:**
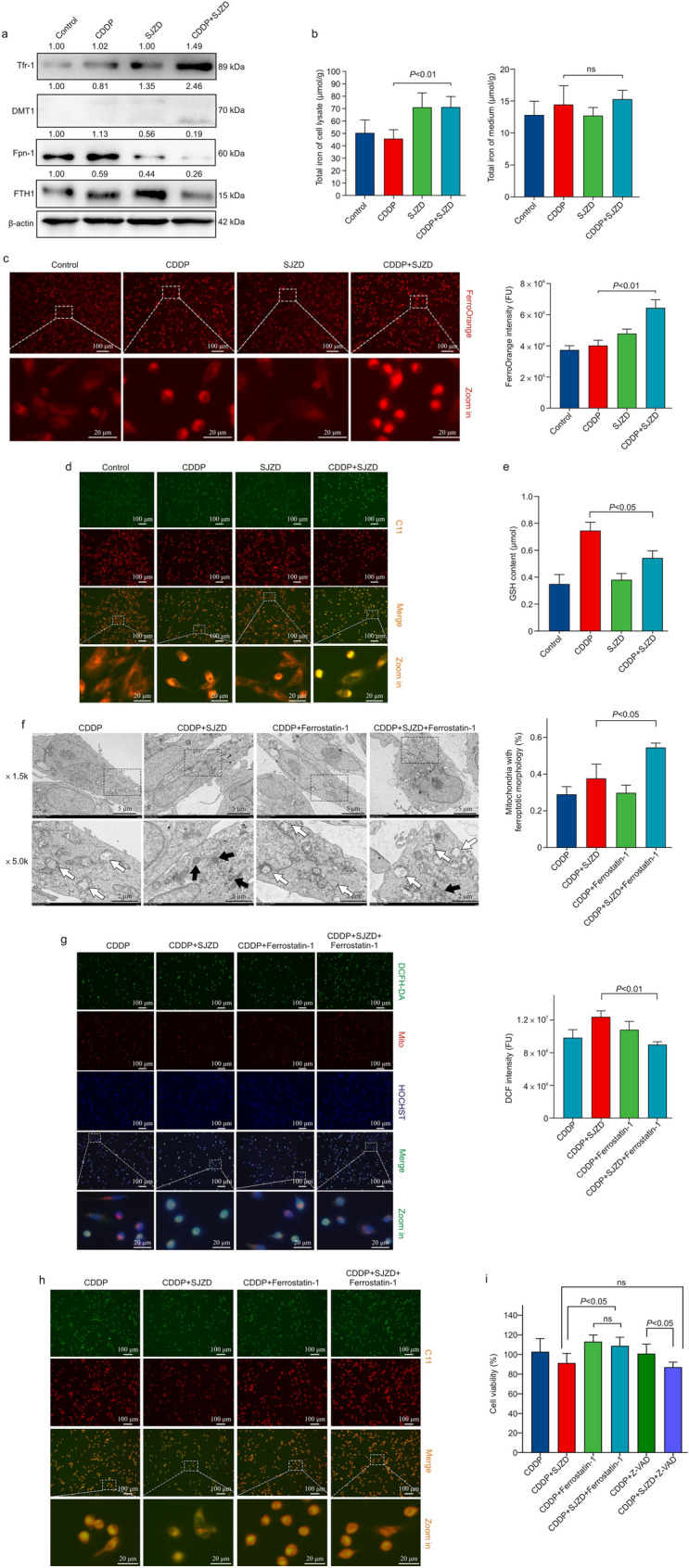
Effects of combined treatment with SJZD and CDDP on iron metabolism and ferroptosis in glutamine-deprived A549/DDP cells. **a** The effect of SJZD + CDDP on the expression of proteins related to iron metabolism in glutamine-deprived A549/DDP cells was detected by WB (n = 3/group). **b** Effect of SJZD + CDDP on total iron in glutamine-deprived A549/DDP cell medium or lysate was quantified via the colorimetric method (n = 3/group). **c** A FerroOrange fluorescence probe was used to observe the effect of SJZD (34 g/kg) on ferrous iron levels in glutamine-deprived A549/DDP cells under a fluorescence microscope, and the results were quantified using a fluorescence microplate (n = 3/group). **d** C11-BODIPY^581/591^, a lipid peroxidation probe, was used to observe the effect of combined treatment with SJZD (34 g/kg) on lipid peroxidation in A549/DDP cells under glutamine deprivation (n = 3/group). **e** Effect of SJZD + CDDP on GSH levels in A549/DDP cells under glutamine deprivation conditions was determined using a colorimetric method (n = 3/group). **f** Representative TEM images of mitochondrial morphology and quantification of mitochondria with ferroptotic morphology (%) were performed to evaluate the role of ferroptosis after combined treatment with SJZD (34 g/kg) in glutamine-deprived A549/DDP cells, with or without the ferroptosis inhibitor Fer-1 (10 μM; 48 h) (n = 3/group). **g** DCFH-DA, MitoTracker, and Hoechst 33,342 probes were used to visualize whether mitochondrial ROS accumulation induced by SJZD in glutamine-deprived A549/DDP cells could be rescued by Fer-1 treatment (34 g/kg), as shown under a fluorescence microscope; the fluorescence intensity was quantified using a fluorescence microplate (n = 3/group). **h** A C^11^-BODIPY^581/59^ probe was used to visualize whether lipid peroxidation in glutamine-deprived A549/DDP cells induced by SJZD in combination treatment (34 g/kg) could be rescued by Fer-1, as shown under a fluorescence microscope (n = 3/group). **i** A CCK-8 assay was performed to confirm whether the cell death induced by the SJZD combination treatment (34 g/kg) could be reversed by Fer-1 (10 μM; 48 h) or Z-VAD-FMK (5 μM; 48 h) in glutamine-deprived A549/DDP cells (n = 3/group). The data represent mean ± SD

Compared with those in the CDDP group, the expression of Tfr-1 and DMT1 and the expression of FPN and FTH1 in the combination group differed (Fig. 6a). Further iron quantification assays revealed conserved total iron levels in the culture medium (*P* > 0.05) but significantly elevated intracellular iron content in cellular homogenates (*P* < 0.01) following combination treatment (Fig. 6b). These observations were corroborated by FerroOrange staining, which revealed increased ferrous iron accumulation in the combination group (*P* < 0.01; Fig. 6c). Iron dysregulation was associated with enhanced lipid peroxidation, as evidenced by elevated C^11^-BODIPY^581/591^ fluorescence (Fig. 6d) and concurrent GSH depletion (*P* < 0.05; Fig. 6e). Ultrastructural analysis via TEM revealed that combination treatment induced typical ferroptotic mitochondrial morphology, characterized by a decrease in the number of mitochondria and a loss of cristae, which were effectively reversed by the ferroptosis inhibitor ferrostatin-1 (Fer-1). Quantitative analysis confirmed that the percentage of mitochondria with a ferroptotic morphology was significantly reversed by Fer-1 (*P* < 0.05; Fig. 6f). Mechanistic investigations demonstrated that Fer-1 completely abrogated mitochondrial ROS accumulation (*P* < 0.01; Fig. 6g) and lipid peroxidation (Fig. 6h), as quantified through DCFH-DA fluorescence and C^11^-BODIPY^581/591^ probes. Crucially, cell viability assays confirmed that Fer-1 (*P* < 0.05) completely reversed the cell death induced by the SJZD combination treatment, whereas Z-VAD-FMK (*P* > 0.05) failed to do so (Fig. 6i). In other words, SJZD combination treatment requires functional ferroptosis pathways to exert cytotoxic effects on A549/DDP cells under glutamine-deprived conditions. Collectively, these investigations establish that SJZD preferentially induces ferroptosis over apoptosis in cisplatin-resistant NSCLC cells, particularly under glutamine-deficient microenvironmental conditions.

### SJZD Inhibits the p62/Keap1/Nrf2 Signaling Pathway in Cisplatin-Resistant NSCLC Under Glutamine Deprivation

**Fig. 7:**
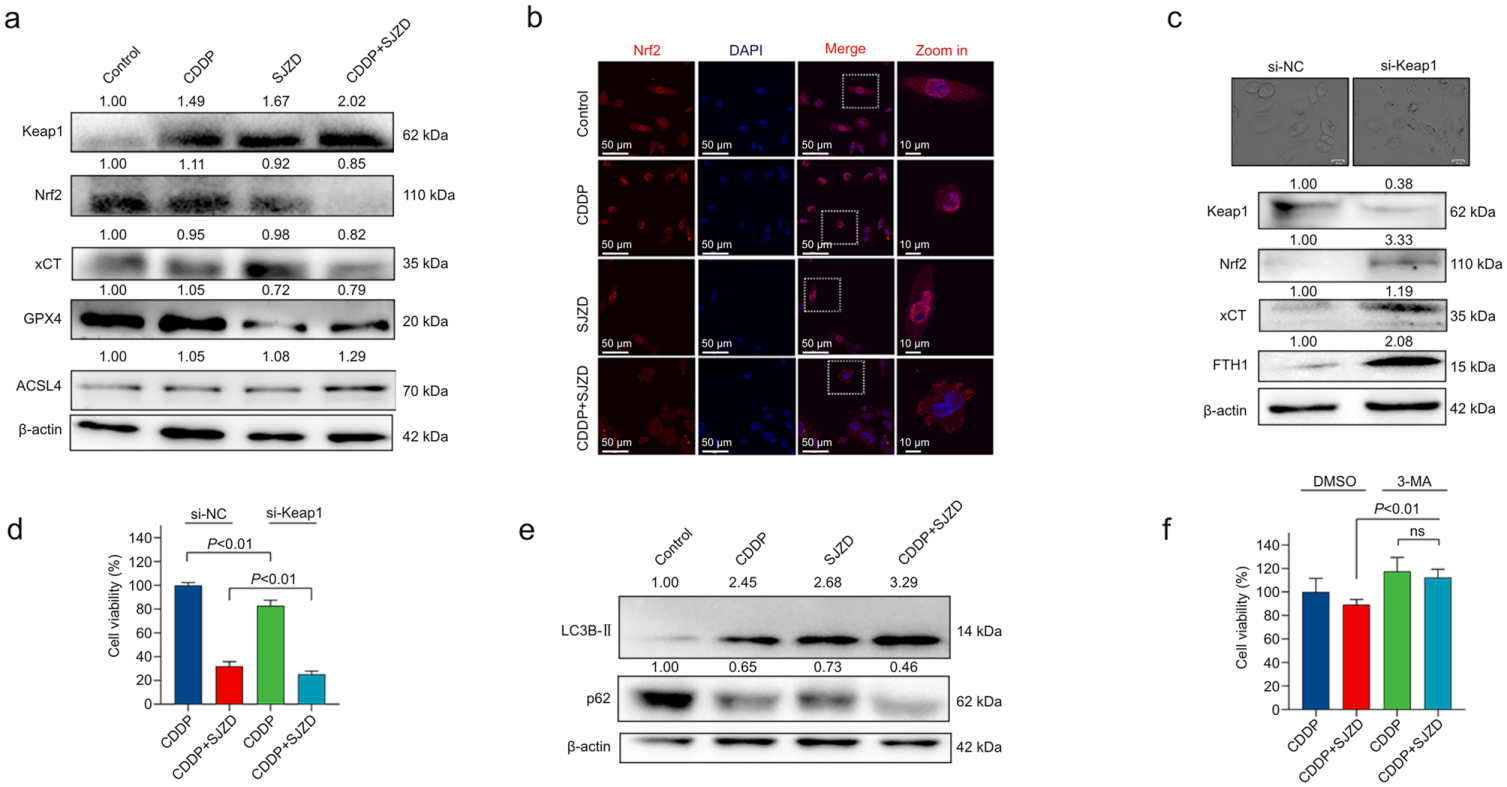
Effects of SJZD + CDDP on autophagy and p62/Keap1/Nrf2 signaling in glutamine-deprived A549/DDP cells. **a** Effects of SJZD + CDDP on Keap1/Nrf2, xCT, GPX4, and ACSL4 protein expression in glutamine-deprived A549/DDP cells were detected by WB (n = 3/group). **b** The effect of SJZD combined with CDDP (34 g/kg) on the nuclear localization of Nrf2 in glutamine-deprived A549/DDP cells was visualized by IF (n = 3/group). **c** The knockdown efficiency of si-Keap1 was verified by WB (n = 3/group). **d** The inhibitory effect of the SJZD + CDDP on A549/DDP cells was monitored using a CCK-8 assay (n = 3/group). **e** The effect of SJZD + CDDP on autophagy-related protein expression in glutamine-deprived A549/DDP cells was detected by WB (n = 3/group). **f** A CCK-8 assay was performed to confirm whether the cell death induced by the SJZD combination treatment (34 g/kg) could be reversed by 3-MA (1 mM, 16 h) in glutamine-deprived A549/DDP cells (n = 3/group). The data represent mean ± SD

Immunoblotting revealed upregulated expression of Keap1 and ACSL4, which was accompanied by the concurrent downregulation of Nrf2, xCT, and GPX4 in the combination group compared with the CDDP group (Fig. 7a). Subsequent immunofluorescence analysis demonstrated reduced nuclear translocation of Nrf2 in combination-treated cells (Fig. 7b). Mechanistically, compared with si-NC, siRNA-mediated *Keap1* silencing (validated by immunoblotting) reversed this regulatory pattern and significantly increased the protein levels of Nrf2, xCT, and FTH (Fig. 7c). Next, CCK-8 assays revealed that knockdown of *Keap1* exacerbated cellular vulnerability, with the viability of glutamine-deprived A549/DDP cells significantly decreased in the si-Keap1 groups under both the CDDP monotherapy (*P* < 0.01) and combination treatment (*P* < 0.01) paradigms (Fig. 7d). Further investigation revealed that compared with CDDP, combination therapy induced autophagic flux activation, characterized by increased LC3B-II conversion and reduced p62/SQSTM1 accumulation (Fig. 7e). Notably, the enhanced cytotoxicity observed in the SJZD + CDDP combination group was abrogated by cotreatment with the autophagy inhibitor 3-methyladenine (3-MA), as evidenced by a significant increase in cell viability (*P* < 0.01). Compared with CDDP alone, SJZD alone failed to further reduce cell viability, demonstrating that autophagy is required for the synergistic effect of SJZD and CDDP (Fig. 7f).

### SJZD Inhibits Tumor Growth in Cisplatin-Resistant NSCLC Xenografts but Shows No In Vivo Toxicity

**Fig. 8:**
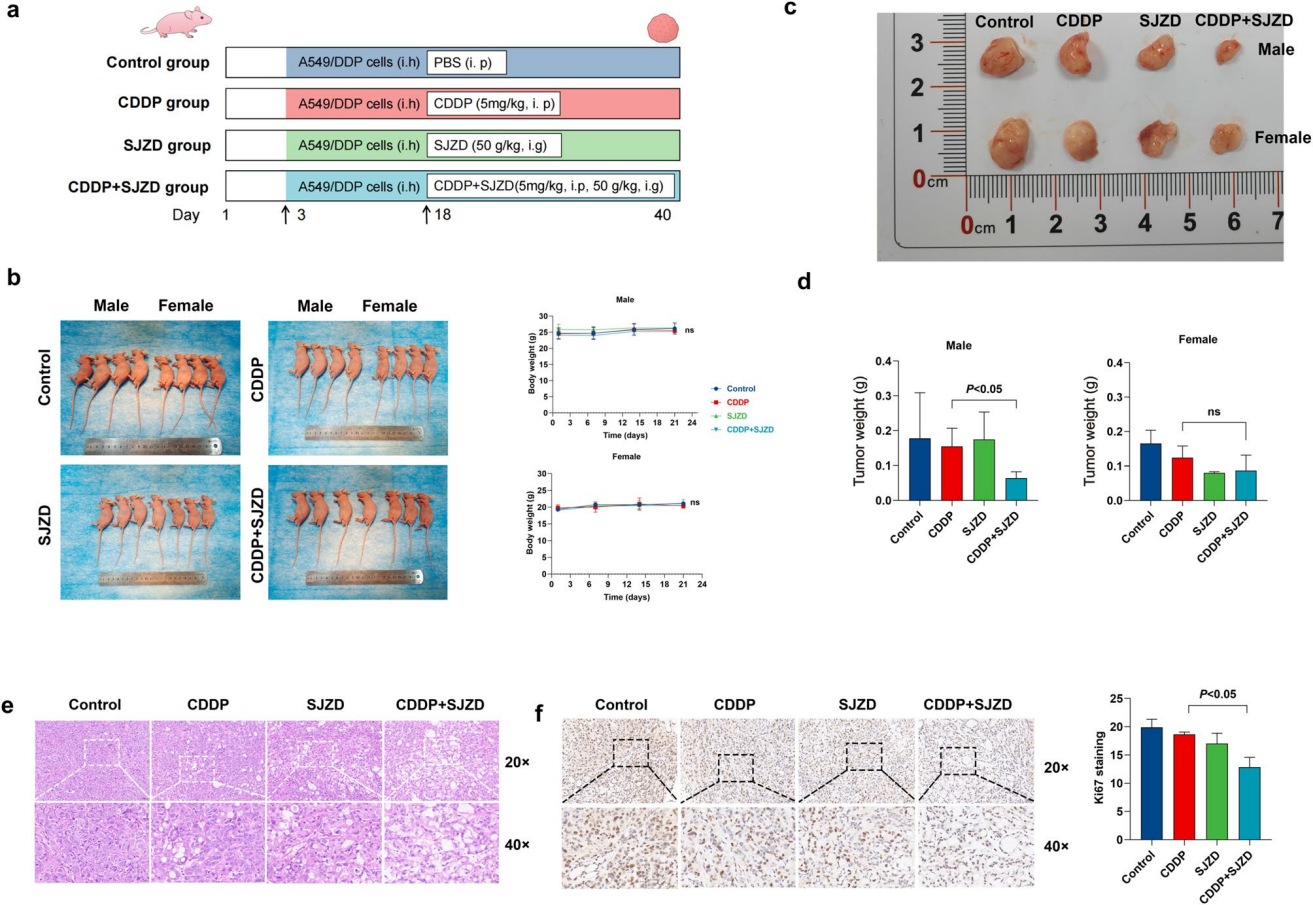
Effects of SJZD combination treatment on cisplatin-resistant NSCLC xenografts in nude mice. **a** Scheme of the experimental design to investigate the effect of SJZD in response to cisplatin-resistant NSCLC. The mice were treated with CDDP (50 mg/kg, once a week for 3 weeks) and/or SJZD (50 g/kg, once a day for 3 weeks) as indicated. **b** Effect of SJZD + CDDP on the body weight of nude mice (n = 4/group). **c, d** Effect of SJZD + CDDP on tumor weight in cisplatin-resistant NSCLC xenografts (n = 4/group). **e** Effect of SJZD combination treatment on histopathology in cisplatin-resistant NSCLC xenografts (n = 3/group). **f** Effect of SJZD combined with CDDP on Ki-67 protein expression in cisplatin-resistant NSCLC xenografts was detected and quantified by IHC (n = 3/group). The data represent mean ± SD

BALB/c nude mice bearing cisplatin-resistant A549/DDP xenografts received SJZD for 21 consecutive days (50 g/kg) daily, as indicated in Fig. 8a. Compared with CDDP monotherapy, the combined intervention resulted in favorable safety profiles, with no significant differences in body weight (*P* > 0.05; Fig. 8b) but elicited a marked reduction in tumor weight (*P* < 0.05; male sex; Fig. 8c/d). HE staining revealed a disorganized tumor structure characterized by reduced cellular cohesion in the combined intervention group (Fig. 8e). Complementary immunohistochemical analysis demonstrated significant attenuation of the Ki-67 proliferation index (*P* < 0.05), confirming the decrease in tumor cell viability following combination therapy (Fig. 8f). Additionally, the organ coefficients (heart, liver, spleen, and lungs) and corresponding histological findings did not significantly differ between the SJZD-treated group and the control group (*P* > 0.05; Fig. S2a–b). Furthermore, the serum levels of alanine aminotransferase (ALT), aspartate aminotransferase (AST), blood urea nitrogen (BUN), and creatinine (Cr) were not significantly different between the two groups (*P* > 0.05; Fig S2c), suggesting that no overt acute organ toxicity occurred at this dose.

### SJZD Induces Ferroptosis and Inhibits the p62/Keap1/Nrf2 Signaling Pathway in Cisplatin-Resistant Xenografts

**Fig. 9:**
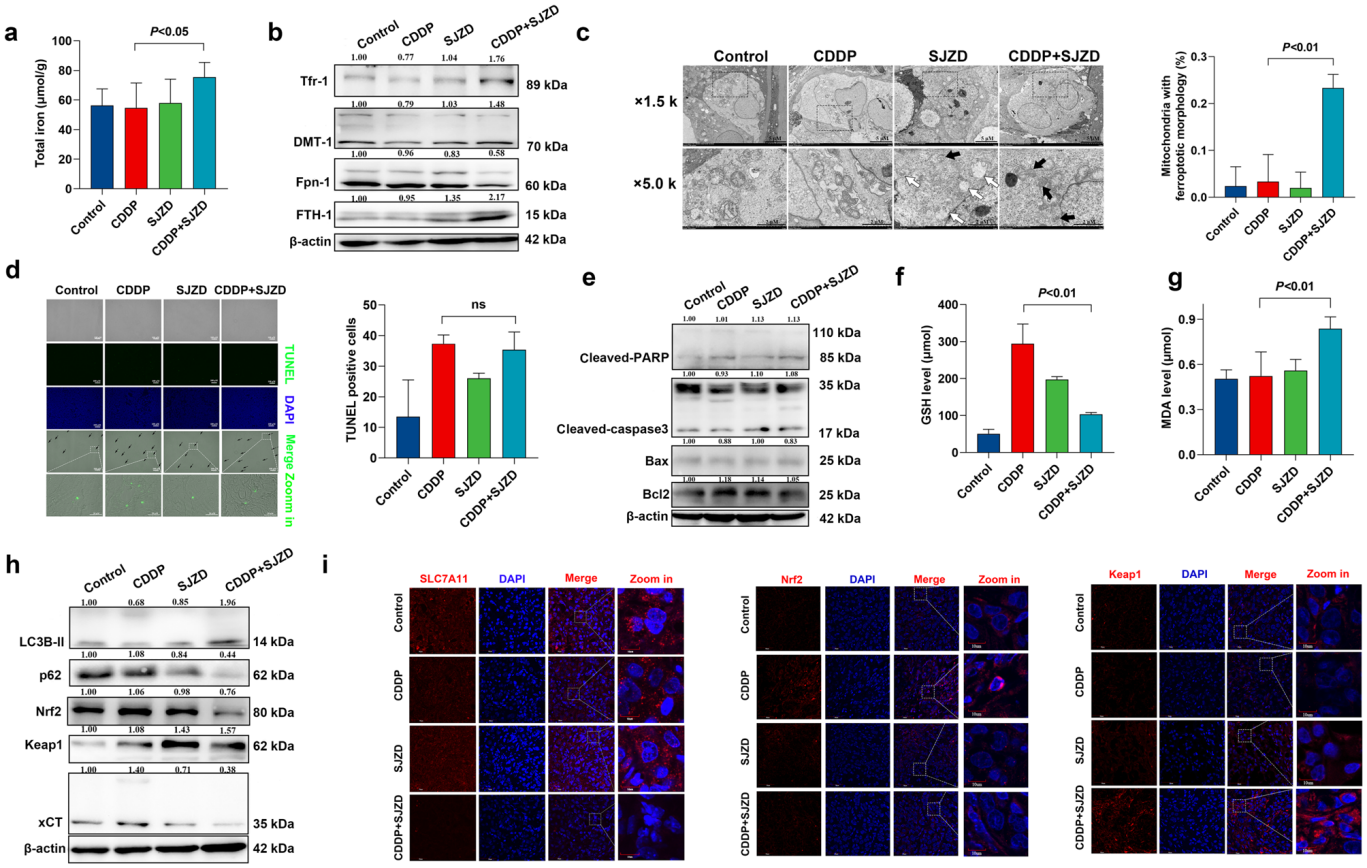
Effects of SJZD combined with CDDP on autophagy and Keap1/Nrf2 signaling in cisplatin-resistant NSCLC xenografts. Effects of SJZD + CDDP on **a** total iron and **b** iron metabolism–related protein levels in xenografts were assessed via the colorimetric method and WB, respectively (n = 3/group). **c** TEM was used to observe whether the SJZD combination induced ferroptosis, and the results were quantified by calculating the percentage of mitochondria exhibiting ferroptotic morphology (white arrows indicate mitochondrial vacuolation and autophagy; black arrows indicate shrunken mitochondria). The effect of the SJZD combination treatment on apoptosis in xenografts was visualized and quantified by **d** TUNEL staining and **e** WB (n = 3/group). Effects of SJZD + CDDP on **f** GSH and **g** MDA levels in xenografts were determined via a colorimetric method (n = 3/group). **h** Effects of SJZD + CDDP on autophagy and Keap1/Nrf2 signaling were detected by WB (n = 3/group).** i** Effects of SJZD + CDDP on the expression of xCT, nuclear localization of Nrf2, and Keap1 in cisplatin-resistant NSCLC xenografts were visualized by IF (n = 3/group). The data represent mean ± SD

Through integrated analytical approaches, we systematically characterized the biological differences between the combination therapy and cisplatin monotherapy groups. Quantitative colorimetric analysis revealed elevated total iron concentrations in the combination group (*P* < 0.05; Fig. 9a), with concordant upregulation of iron metabolism regulators, including Tfr1, DMT1, and FTH1. Notably, FPN expression remained comparable between the treatment groups (Fig. 9b). Subsequent ultrastructural analysis revealed that the percentage of mitochondria with a ferroptotic morphology was significantly greater in the combination group than in the cisplatin control group (*P* < 0.05; Fig. 9c). Quantitative assessment of apoptotic activity through TUNEL staining revealed no intergroup disparity in the cell death rate (*P* > 0.05; Fig. 9d), which was supported by the unchanged proteomic profiles of the apoptosis-related markers cleaved PARP, cleaved caspase-3, Bcl-2 and Bax (Fig. 9e). Crucially, biochemical evaluation revealed significant accumulation of lipid peroxidation byproducts (MDA) coupled with depletion of GSH in the combination group compared with the cisplatin-treated groups (all *P* < 0.01; Fig. 9f–g). Western blotting analysis revealed differential expression of autophagy and oxidative stress mediators, including upregulation of LC3B-II and Keap1 and downregulation of p62, Nrf2, and xCT, in the combination group compared with those in the cisplatin-treated groups (Fig. 9h). Immunofluorescence colocalization studies corroborated these molecular dynamics, confirming that Keap1 expression increased with concomitant suppression of Nrf2 and xCT expression (Fig. 9i).

## Discussions

Following systemic absorption, distribution, and biotransformation, rat serum includes both SJZD-derived phytochemicals and their regulated metabolites. Together, these components form the pharmacological foundation for therapeutic efficacy. Shang et al. administered SJZD to rats at tenfold the clinical adult dose to investigate its major metabolites in serum, confirming that bioactive components (ginsenosides and glycyrrhizin) are present at such a high dose [23]. Our drug-containing serum preparation method followed a protocol consistent with that in the literature. Our serum pharmacochemical profiling also demonstrated that SJZD modulates glutamine metabolic pathways, which is consistent with prior evidence that SJZD reverses gefitinib resistance by targeting glutamine in lung adenocarcinoma [24]. However, the characteristics of cisplatin-resistant NSCLC cells are distinct from those of their parental cells. As we previously reported, these resistant NSCLC cells display an adaptive response that balances metabolic stability and plasticity, allowing them to survive under stress induced by glutamine starvation and overcome metabolic reprogramming [10].

To elucidate the therapeutic mechanisms of SJZD beyond glutamine metabolism, cisplatin-resistant NSCLC cells were subjected to glutamine starvation in accordance with our previous study [10], which enabled systematic exploration of the role of SJZD in counteracting the adaptive response under metabolic stress.

### SJZD Counteracts Metabolic Adaptation by Disrupting Iron Metabolism, leading to the Attenuation of TCA Cycle Metabolites and the Subsequent Induction of Oxidative Stress in Cisplatin-Resistant NSCLC

Alterations in TCA cycle metabolites reflect dynamic changes in cellular metabolic reprogramming [25]. In the present study, targeted metabolomics revealed reduced levels of cis-aconitate and fumarate in the SJZD combination–treated groups. These core intermediates interconnect within the TCA cycle, governing energy metabolism, biosynthetic pathways, and redox homeostasis. Cis-aconitate undergoes iron-sulfur cluster (ISC)-dependent dehydration to aconitate via aconitase catalysis. Crucially, mitochondrial respiration requires ISC-containing proteins to facilitate electron transport and OXPHOS [26]. Owing to the multifaceted role of iron in ISC biogenesis, its dysregulation may impair ISC function and promote oxidative stress [27]. We demonstrated that the SJZD combination treatment induced oxidative stress in glutamine-deprived A549/DDP cells. Notably, this effect occurred despite the absence of significant changes in NADPH levels, while marked reductions in fumarate and cis-aconitate concentrations were observed. Beyond their canonical roles in energy metabolism, cis-aconitate and fumarate have emerged as key regulators of redox homeostasis and antioxidant defense systems. Specifically, cis-aconitate serves as the precursor to itaconate synthesis, which exerts potent antioxidant effects by activating the Nrf2 pathway. Similarly, fumarate exhibits immunomodulatory and antioxidant properties [28]. The dynamic interplay among these metabolites under oxidative stress is further supported by evidence from sepsis models, in which increased levels of cis-aconitate and itaconate, accompanied by reduced fumarate, correlate with redox imbalance [29]. These findings suggest that SJZD potentially disrupts metabolic adaptation mechanisms in cisplatin-resistant NSCLC cells during glutamine deprivation. On the basis of these findings, we hypothesize that SJZD interferes with iron metabolism, leading to attenuated TCA cycle intermediates and, consequently, oxidative stress under glutamine deprivation.

Dysregulated iron homeostasis, characterized by elevated Tfr-1 expression and reduced FTH1 expression [30], was confirmed in glutamine-deprived A549/DDP cells following SJZD combination treatment. Accumulating evidence has established that iron dysregulation drives oxidative stress, lipid peroxidation, and ferroptosis [30, 31]. Given the established link between ferroptosis and cellular energy metabolism, we used pharmacological inhibitors to confirm the effects of SJZD: the apoptosis inhibitor Z-VAD-FMK and the ferroptosis inhibitor Fer-1. Crucially, Fer-1 (but not Z-VAD-FMK) reversed SJZD-mediated growth inhibition. Notably, the SJZD combination suppressed cell viability even in response to Z-VAD-FMK cotreatment in glutamine-deprived cisplatin-resistant NSCLC cells. Additionally, Fer-1 specifically rescued mitochondrial ROS accumulation, lipid peroxidation, and mitochondrial condensation and increased membrane density, which collectively demonstrated that SJZD triggered ferroptosis in glutamine-deprived cisplatin-resistant NSCLC cells.

### SJZD Counteracts Metabolic Adaptation by Inducing Ferroptosis via Inhibition of the p62/Keap1/Nrf2 Pathway in Cisplatin-Resistant NSCLC

Lung tumors exist within a highly oxidized microenvironment [32]. Zhao et al. demonstrated that Nrf2 silencing (si-Nrf2) promotes alcohol-induced ferroptosis in HepG2 cells, whereas Nrf2 transcriptional activation confers ferroptosis resistance [33]. Notably, Singh et al. revealed that si-Nrf2 sensitizes A549 tumor cells to carboplatin by downregulating multidrug resistance protein (MRP) expression, whereas Keap1 knockdown (si-Keap1) upregulates MRP1/2 in human bronchial epithelium cells [34]. These findings collectively indicate that constitutive Nrf2 activation drives chemoresistance. Consequently, inhibition of Nrf2 presents a promising therapeutic approach for overcoming drug resistance in NSCLC. Mechanistically, Keap1 functions as a molecular switch that regulates Nrf2-mediated antioxidant responses under cellular oxidative stress conditions [35]. In *Keap1*-mutated lung adenocarcinomas (LUADs), elevated antioxidant capacity, primarily driven by Nrf2-dependent activation of antioxidant response elements (AREs), is associated with poor clinical outcomes. Romero et al. further reported that Keap1 deficiency increases the susceptibility of *Kras*-mutant LUADs to glutaminase inhibitors (BPTES/CB-839) [36, 37]. Complementary work by Mitsuishi et al. demonstrated that Nrf2 activation in Keap1-deficient LUAD promotes glutamine anabolism and accelerates tumor proliferation [38]. Thus, targeting glutamine metabolism has emerged as a promising strategy for *Kras/Keap1*-mutant NSCLC tumors.

The A549 cell line—a representative *Kras*-driven LUAD model—exhibits loss of heterozygosity (LOH) at the *Keap1* locus (19p13.2), resulting in a G333C mutation within the Keap1 Kelch domain. This mutation partially disrupts Keap1-mediated regulation of Nrf2, leading to constitutive Nrf2 activation in A549 cells [39]. In the present study, Keap1 knockdown (si-Keap1) further upregulated Nrf2 and its associated antioxidant proteins in cisplatin-resistant A549/DDP cells. Notably, despite the preexisting *Keap1* mutation, si-Keap1 retained its role as a molecular switch regulating Nrf2 activity, which is consistent with the findings of Tirumalai et al. [40].

### Limitations and Prospects

Despite these promising results, several limitations of this study warrant consideration. Multiple studies have reported the antitumor effects of SJZD, with effective doses varying considerably across different experimental models, ranging from 8.58 to 38.60 g/kg, making it difficult to determine a standardized dosage for antitumor studies [19, 41–43]. On the basis of the positive results from our *in vitro* SJZD-medicated serum experiments (34 g/kg in rats) and the method established by Shang et al. [23], we subsequently used the 10 × clinical equivalent dose (50 g/kg in mice) for *in vivo* validation. Although we conducted preliminary safety assessments by evaluating organ coefficients, HE staining, and liver/kidney functions, which revealed no significant abnormalities at the 50 g/kg dose under the current experimental conditions, these data are insufficient to comprehensively assess long-term organ toxicity. Therefore, dedicated chronic toxicity studies are warranted to fully establish the safety profile of SJZD and support its clinical feasibility. Additionally, although si-Keap1 increased Nrf2 expression, it also enhanced cellular sensitivity to both cisplatin monotherapy and SJZD combination treatment. Zhang et al. reported analogous biphasic effects in A549 cells, in which moderate Keap1 inactivation promoted Nrf2-mediated chemoprotection, whereas complete Keap1 loss activated PPARγ expression, thereby inhibiting proliferation and counteracting Nrf2-driven chemoresistance [44].

**Fig. 10:**
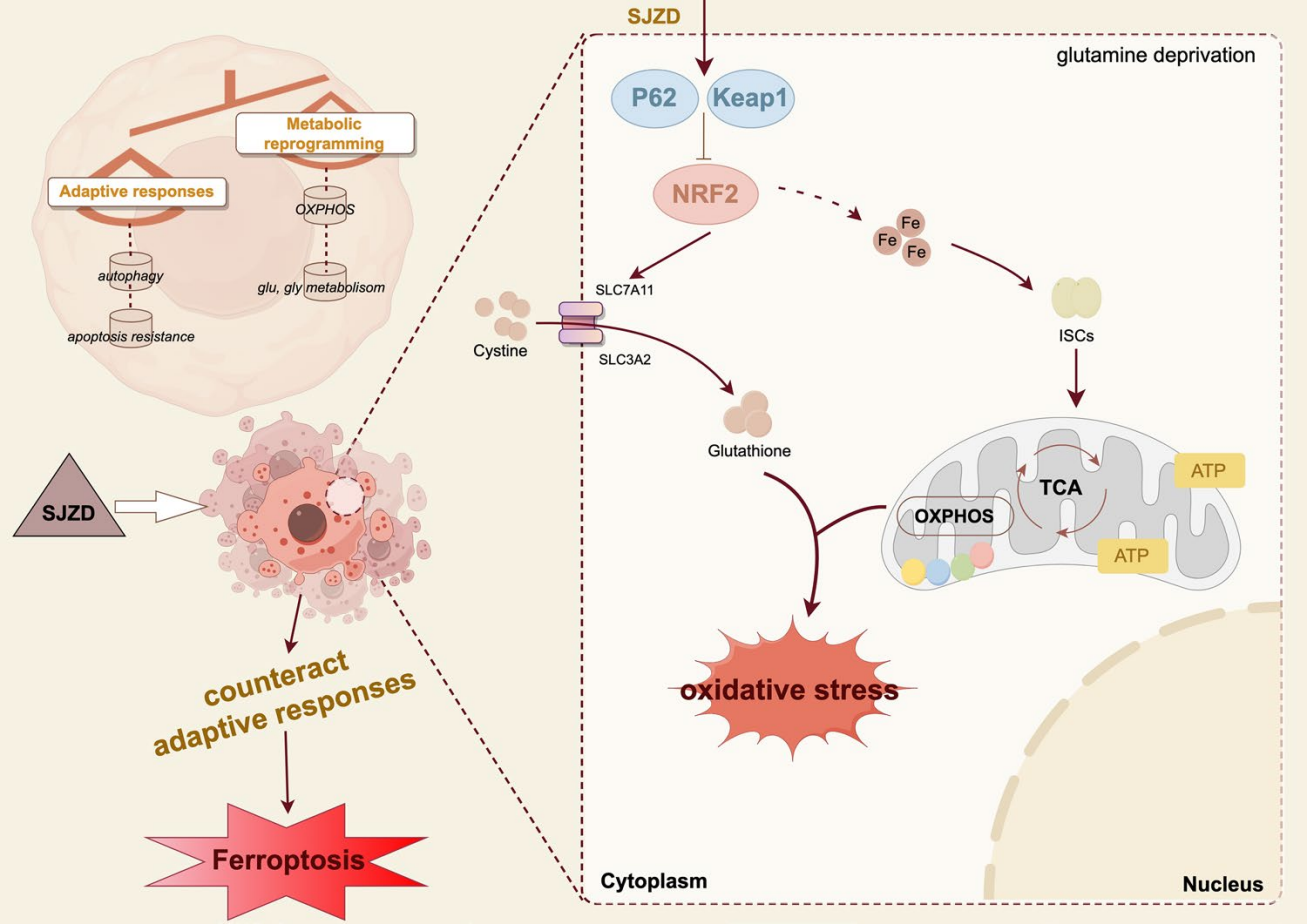
Schematic diagram depicting the mechanism of SJZD in cisplatin-resistant NSCLC. Although pharmacological targeting of glutamine metabolism represents a promising anticancer strategy, its efficacy is frequently limited by adaptive response in cisplatin-resistant NSCLC, as previously established (upper left panel). SJZD, a canonical TCM formulation, effectively counteracts this adaptation through (i) induction of oxidative stress; (ii) attenuation of TCA cycle flux; (iii) intracellular ferrous iron accumulation; and (iv) subsequent lipid peroxidation, collectively triggering p62/Keap1/Nrf2 axis-suppressed ferroptosis. The figure is generated by FigDraw

Collectively, these data demonstrate the dual role of the Keap1/Nrf2 axis in modulating chemoresistance in NSCLC (Fig. 10). This evidence supports the potential of combined Keap1/Nrf2 coinhibition—rather than Nrf2 blockade alone—as a superior antitumor strategy.

## Conclusion

SJZD counteracts metabolic adaptation via ferroptosis by inhibiting the p62/Keap1/Nrf2 pathway in cisplatin-resistant NSCLC. These findings provide a solid basis for counteracting the adaptive response in cisplatin-resistant NSCLC for therapeutic benefit. Therefore, SJZD may improve treatment outcomes in patients with cisplatin-resistant NSCLC.

## Supplementary Information

Below is the link to the electronic supplementary material.

Supplementary file1 (DOC 10409 KB)

## Data Availability

The datasets and materials used and/or analyzed in the current study are available from the corresponding author on reasonable request.
