# Supplementary material for: Sijunzi Decoction Reverses Metabolic Adaptation and Induces Ferroptosis in Cisplatin-Resistant Non–small Cell Lung Cancer: An Integrative Metabolomics–Pharmacology Analysis
Source: Curr Med Sci. 2026 May 13;46(3):739–59. doi: 10.1007/s11596-026-00203-x (PMC13314822; doi:10.1007/s11596-026-00203-x)
Supplement: Supplementary file 1 — Supplementary file1 (DOC 10409 KB) [file 11596_2026_203_MOESM1_ESM.doc]

**Supplementary Materials**


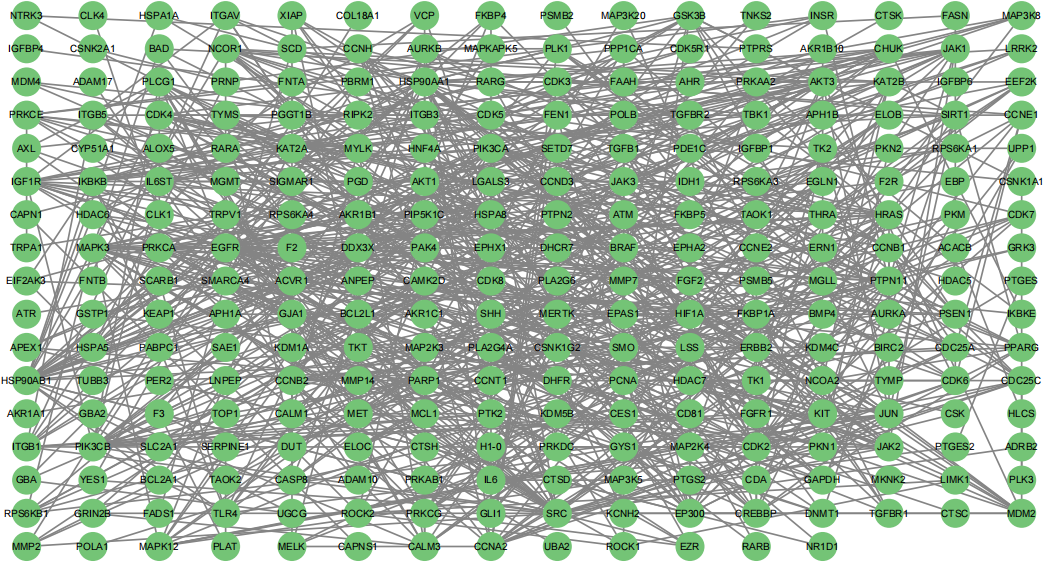


**Fig. S1** A primary PPI network containing 269 targets interconnected by 739 edges


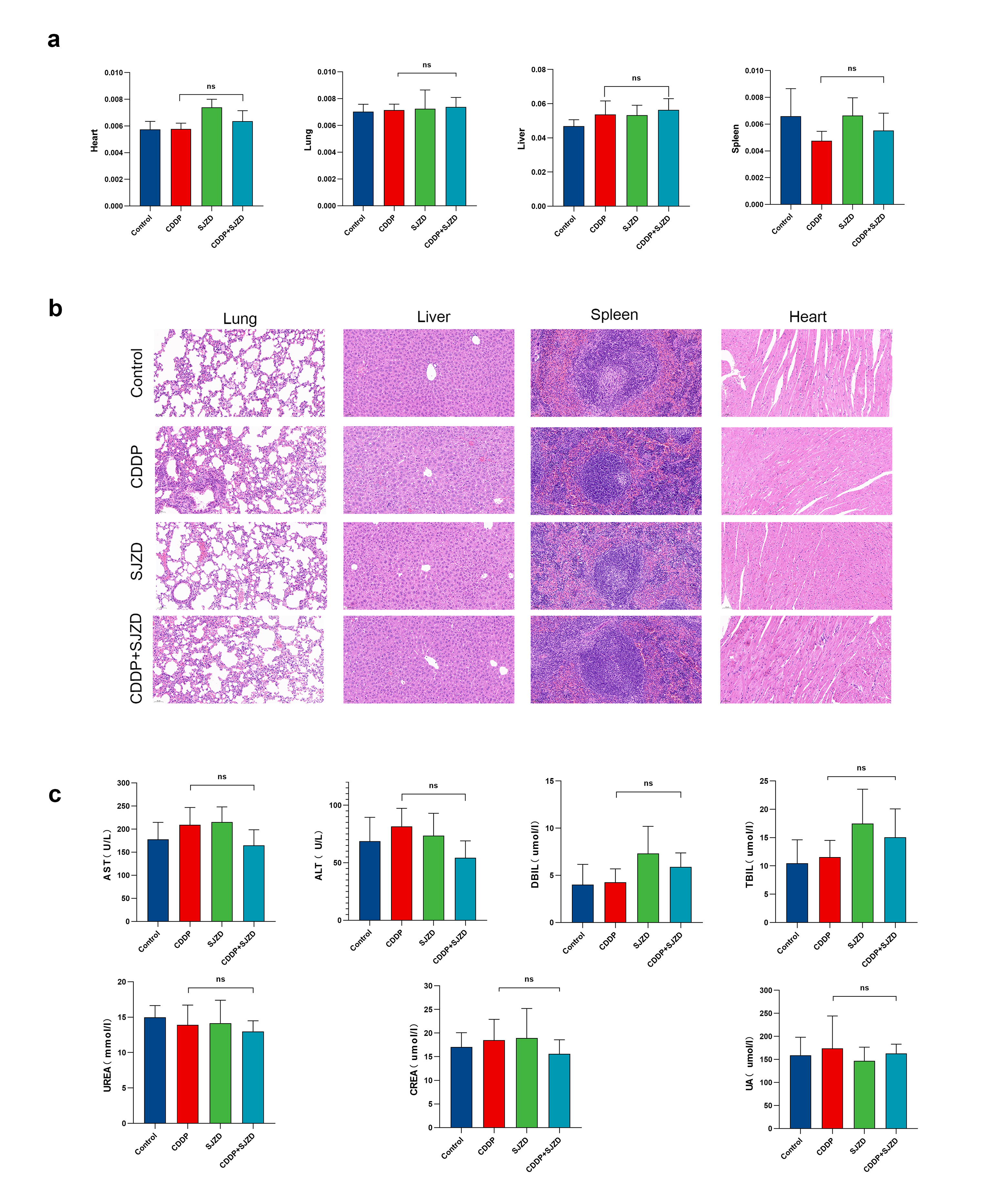


**Fig. S2** Organ coefficients and serum biochemical parameters

**a** Effects of SJZD combination medication on organ coefficients (heart, liver, spleen, and lung) in mice (n = 4/group). **b** Effects of SJZD combination medication on histopathology (heart, liver, spleen, and lung) in cisplatin-resistant NSCLC xenografts (n = 3/group). **c** Effects of SJZD combination medication on liver/kidney function in cisplatin-resistant NSCLC xenografts (n = 4/group). The data represent the mean ± SD of at least three independent experiments. Compared to CDDPgroup, ^*^*P* < 0.05.

| ID | NameEN | Formula | mzmed | rtmed | ppm | type | ms2Adduct | **blood_level** |
| --- | --- | --- | --- | --- | --- | --- | --- | --- |
| SJZDLI01 | 8'-Episesaminone | C20H18O7 | 371.1115 | 376.2 | 2.9 | POS | [M+H]+ | I |
| SJZDLI02 | 8-Gingerol | C19H30O4 | 345.2011 | 110.1 | 3.1 | POS | [M+Na]+ | I |
| SJZDLI03 | Angeloylgomisin H | C28H36O8 | 499.2232 | 41.3 | 13.9 | POS | M-H | I |
| SJZDLI04 | (+)-Costunolide | C15H20O2 | 233.1534 | 219 | 0.9 | POS | [M+H]+ | I |
| SJZDLI05 | (R)-1-((4,8-dimethoxyfuro[2,3-b]quinolin-7-yl)oxy)-3-methylbutane-2,3-diol | C18H21NO6 | 370.1357 | 358.9 | 15.4 | POS | [M+Na]+ | I |
| SJZDLI06 | [1a-(hydroxymethyl)-2-[3,4,5-trihydroxy-6-(hydroxymethyl)oxan-2-yl]oxy-2,5a,6,6a-tetrahydro-1bH-oxireno[5,6]cyclopenta[1,3-c]pyran-6-yl] 3,4-dimethoxybenzoate | C24H30O13 | 549.1593 | 279.5 | 4.7 | POS | [M+Na]+ | I |
| SJZDLI07 | 5,7-dihydroxy-2-(4-hydroxyphenyl)-6-[3,4,5-trihydroxy-6-(hydroxymethyl)tetrahydropyran-2-yl]-8-(3,4,5-trihydroxytetrahydropyran-2-yl)chromen-4-one | C26H28O14 | 565.1542 | 270.9 | 1.8 | POS | [M+H]+ | I |
| SJZDLI08 | 5,7-dihydroxy-2-(4-hydroxyphenyl)-8-[3,4,5-trihydroxy-6-(hydroxymethyl)tetrahydropyran-2-yl]-6-(3,4,5-trihydroxytetrahydropyran-2-yl)chromen-4-one | C26H28O14 | 565.1542 | 270.9 | 1.8 | POS | [M+H]+ | I |
| SJZDLI09 | 5,7-dihydroxy-2-[4-[3,4,5-trihydroxy-6-(hydroxymethyl)tetrahydropyran-2-yl]oxyphenyl]chroman-4-one | C21H22O10 | 435.128 | 300.2 | 1.3 | POS | [M+H]+ | I |
| SJZDLI10 | 5-hydroxy-2-(4-hydroxyphenyl)-7-[(2S,3R,4S,5S,6R)-3,4,5-trihydroxy-6-[[(2R,3R,4R,5R,6S)-3,4,5-trihydroxy-6-methyloxan-2-yl]oxymethyl]oxan-2-yl]oxy-2,3-dihydrochromen-4-one | C27H32O14 | 598.2121 | 243 | 1.5 | POS | [M+NH4]+ | I |
| SJZDLI11 | 5-methyl-4-[(2S,3R,4S,5S,6R)-3,4,5-trihydroxy-6-(hydroxymethyl)tetrahydropyran-2-yl]oxy-chromen-2-one | C16H18O8 | 339.1065 | 259.5 | 2.9 | POS | [M+H]+ | I |
| SJZDLI12 | gamma-mangostin | C23H24O6 | 435.1179 | 282.4 | 5.8 | POS | [M+K]+ | I |
| SJZDLI13 | Glycyrrhisoflavone | C20H18O6 | 355.1165 | 413 | 3.2 | POS | [M+H]+ | I |
| SJZDLI14 | Glyurallin_A即Glyurallin A | C21H20O5 | 353.1373 | 458.8 | 3 | POS | [M+H]+ | I |
| SJZDLI15 | Apparicine | C18H20N2 | 265.1751 | 40.7 | 19.3 | POS | [M+H]+ | I |
| SJZDLI16 | Atractylenolide I | C15H18O2 | 231.1372 | 428.4 | 3.1 | POS | [M+H]+ | I |
| SJZDLI17 | beta.-Amyrenonol | C30H48O2 | 441.3718 | 339.1 | 2 | POS | [M+H]+ | I |
| SJZDLI18 | Cassiaoccidentalin_B | C27H28O14 | 577.1542 | 296.6 | 1.7 | POS | [M+H]+ | I |
| SJZDLI19 | Daidzin | C21H20O9 | 417.117 | 284.5 | 0 | POS | [M+H]+ | I |
| SJZDLI20 | Enoxolone | C30H46O4 | 471.3454 | 504 | 0.1 | POS | [M+H]+ | I |
| SJZDLI21 | Erosone | C20H16O6 | 353.1011 | 375.8 | 2.5 | POS | [M+H]+ | I |
| SJZDLI22 | Fraxetin | C10H8O5 | 207.0298 | 269.6 | 0.6 | NEG | [M-H]- | I |
| SJZDLI23 | Ponganone_V | C22H22O6 | 383.1478 | 428.7 | 3 | POS | [M+H]+ | I |
| SJZDLI24 | Icariin | C33H40O15 | 721.1979 | 305 | 2.9 | NEG | [M+FA-H]- | I |
| SJZDLI25 | Isoanhydroicaritin | C21H20O6 | 369.1321 | 406.1 | 2.8 | POS | [M+H]+ | I |
| SJZDLI26 | Jasmine lactone | C10H16O2 | 169.1218 | 269.2 | 3 | POS | [M+H]+ | I |
| SJZDLI27 | Malaccol | C20H16O7 | 369.096 | 368.5 | 2.4 | POS | [M+H]+ | I |
| SJZDLI28 | Marrubiin | C20H28O4 | 350.2315 | 350.9 | 3 | POS | [M+NH4]+ | I |
| SJZDLI29 | Matricarin | C17H20O5 | 305.1374 | 355.5 | 3.3 | POS | [M+H]+ | I |
| SJZDLI30 | Neoacrimarine_E | C35H35NO9 | 614.244 | 291.9 | 9 | POS | [M+H]+ | I |
| SJZDLI31 | Nerolidol | C15H26O | 245.1854 | 211.5 | 21.9 | POS | [M+Na]+ | I |
| SJZDLI32 | Nystose | C24H42O21 | 665.2145 | 61.1 | 0.1 | NEG | [M-H]- | I |
| SJZDLI33 | Patchoulenone | C15H22O | 219.1737 | 299.6 | 3.1 | POS | [M+H]+ | I |
| SJZDLI34 | Picraquassioside_A | C18H22O10 | 399.1332 | 404.9 | 11.4 | POS | [M+H]+ | I |
| SJZDLI35 | Pteryxin | C21H22O7 | 387.1433 | 339.9 | 8.5 | POS | [M+H]+ | I |
| SJZDLI36 | Purpurin | C14H8O5 | 255.0296 | 282.1 | 1.3 | NEG | [M-H]- | I |
| SJZDLI37 | Rubelloside A | C42H66O14 | 793.4375 | 429.6 | 0.6 | NEG | [M-H]- | I |
| SJZDLI38 | Saponarin | C27H30O15 | 575.1408 | 310.9 | 0.5 | NEG | [M-H2O-H]- | I |
| SJZDLI39 | Schaftoside | C26H28O14 | 565.1542 | 270.9 | 1.8 | POS | [M+H]+ | I |
| SJZDLI40 | Scropolioside D | C34H42O17 | 767.246 | 69.7 | 7.8 | NEG | [M+FA-H]- | I |
| SJZDLI41 | Semilicoisoflavone B | C20H16O6 | 353.1008 | 417.2 | 3.3 | POS | [M+H]+ | I |
| SJZDLI42 | Smyrindioloside | C20H24O10 | 447.1177 | 322.6 | 5.2 | POS | [M+Na]+ | I |
| SJZDLI43 | Vicenin 3 | C26H28O14 | 565.1542 | 270.9 | 1.8 | POS | [M+H]+ | I |
| SJZDLI44 | Protopine | C20H19NO5 | 354.1406 | 420.2 | 13.2 | POS | [M+H]+ | I |
| SJZDLI45 | Prunin | C21H22O10 | 435.128 | 300.2 | 1.3 | POS | [M+H]+ | I |
| SJZDLI46 | Psoralidin_oxide | C20H16O6 | 353.1009 | 430.7 | 3 | POS | [M+H]+ | I |
| SJZDLI47 | methyl chlorogenate | C17H20O9 | 369.1171 | 260.7 | 0.3 | POS | [M+H]+ | I |
| SJZDLI48 | 5-hydroxy-2-(4-hydroxyphenyl)-7-[(2S,3R,4S,5S,6R)-3,4,5-trihydroxy-6-[[(2R,3R,4R,5R,6S)-3,4,5-trihydroxy-6-methyloxan-2-yl]oxymethyl]oxan-2-yl]oxy-2,3-dihydrochromen-4-one | C27H32O14 | 598.2121 | 243 | 1.5 | POS | [M+NH4]+ | I |
| SJZDLI49 | Matricarin | C17H20O5 | 305.1374 | 355.5 | 3.3 | POS | [M+H]+ | I |
| SJZDLI50 | Icariin | C33H40O15 | 721.1979 | 305 | 2.9 | NEG | [M+FA-H]- | I |
| SJZDLI51 | Scropolioside D | C34H42O17 | 767.246 | 69.7 | 7.8 | NEG | [M+FA-H]- | I |
| SJZDLI52 | Atractyloside potassium salt | C30H44O16S2.2K | 725.2087 | 323.5 | 8.9 | NEG | [M+H]+ | I |
| SJZDLI53 | Angeloylgomisin H | C28H36O8 | 499.2232 | 41.3 | 13.9 | POS | M-H | I |
| SJZDLI54 | Picraquassioside_A | C18H22O10 | 399.1332 | 404.9 | 11.4 | POS | [M+H]+ | I |
| SJZDLI55 | Apparicine | C18H20N2 | 265.1751 | 40.7 | 19.3 | POS | [M+H]+ | I |

**Table S1 55 blood-entry prototype components obtained from LC-MS-based untargeted metabolomics profiling.** Following comprehensive metabolomic characterization of SJZD extracts, SJZD-medicated serum, and NS-medicated serum, integrated qualitative-quantitative approaches identified 55 bioavailable prototype phytochemicals exhibiting systemic absorption (In the last column, level I represents for prototype phytochemicals exhibiting systemic absorption; level II represents for potential prototype phytochemicals exhibiting systemic absorption; level III represents for metabolites of potential prototype phytochemicals exhibiting systemic absorption).

| **Annotation** | | | | | **DE Statistic** | | | | **FPKM** | | | | | | | |
| --- | --- | --- | --- | --- | --- | --- | --- | --- | --- | --- | --- | --- | --- | --- | --- | --- |
| **Track_id** | **Gene_Name** | **Locus** | **Strand** | **Gene_Type** | **log2FC** | **Fold_Change** | **p_value** | **q_value** | **Treatment_FPKM** | **Control_FPKM** | **A549-1** | **A549-2** | **A549-3** | **A549-cis-1** | **A549-cis-2** | **A549-cis-3** |
| ENSG00000244734.3_2 | HBB | chr11:5246694-5250625 | - | protein_coding | -6.628809656 | 0.01010484 | 2.73398E-08 | 7.11431E-05 | 0.036741698 | 6.665551354 | 106.9476513 | 95.11319307 | 99.83155913 | 0.059516534 | 0.018763369 | 0 |
| ENSG00000206172.8_2 | HBA1 | chr16:226679-227521 | + | protein_coding | -6.5413404 | 0.010736441 | 1.82075E-06 | 0.000152873 | 0.442778652 | 6.984119053 | 131.2220085 | 117.8918784 | 128.0720018 | 0.561271953 | 0.442209008 | 0.115224253 |
| ENSG00000188536.12_2 | HBA2 | chr16:222846-223709 | + | protein_coding | -6.232500253 | 0.013299352 | 4.19945E-07 | 0.000113454 | 0.280056232 | 6.512556485 | 96.75079496 | 82.5993355 | 92.13253574 | 0.309294794 | 0.247191231 | 0.096340579 |
| ENSG00000178860.8_2 | MSC | chr8:72753784-72756703 | - | protein_coding | -5.070670092 | 0.029756113 | 6.20623E-08 | 7.11431E-05 | 0 | 5.070670092 | 30.79505168 | 32.25200293 | 34.89996639 | 0 | 0 | 0 |
| ENSG00000130066.16_2 | SAT1 | chrX:23801290-23804343 | + | protein_coding | -4.631253276 | 0.040350958 | 8.03302E-07 | 0.000125844 | 3.000312958 | 7.631566234 | 189.7063737 | 214.6965076 | 188.5757268 | 7.690821062 | 6.911437528 | 6.451374288 |
| ENSG00000147408.14_3 | CSGALNACT1 | chr8:19261672-19615540 | - | protein_coding | -4.293525028 | 0.050994129 | 9.57922E-07 | 0.000132018 | 1.029581198 | 5.323106226 | 40.75179745 | 41.14040323 | 35.46448365 | 1.196600483 | 0.954493102 | 0.981614876 |
| ENSG00000138166.5_2 | DUSP5 | chr10:112257596-112271302 | + | protein_coding | -4.197490555 | 0.054504133 | 1.78021E-07 | 0.000102579 | 2.234012262 | 6.431502817 | 88.04375385 | 80.43547369 | 87.67653358 | 3.53222191 | 3.929759602 | 3.65990644 |
| ENSG00000179715.12_3 | PCED1B | chr12:47473386-47630445 | + | protein_coding | -4.046985044 | 0.060497316 | 1.35041E-06 | 0.000136071 | 1.648531329 | 5.695516373 | 49.6250814 | 50.27061954 | 52.62033309 | 2.504248989 | 2.061383949 | 1.872495646 |
| ENSG00000130513.6_3 | GDF15 | chr19:18485541-18499986 | + | protein_coding | -4.031051396 | 0.061169174 | 8.35446E-07 | 0.000126872 | 3.570061046 | 7.601112442 | 179.7431442 | 190.5798984 | 210.3862647 | 11.4307287 | 10.26117741 | 10.9675705 |
| ENSG00000088002.11_2 | SULT2B1 | chr19:49055332-49102682 | + | protein_coding | -3.990052662 | 0.062932425 | 1.62847E-06 | 0.000144573 | 0.156189384 | 4.146242046 | 17.07385313 | 16.05161137 | 17.01413863 | 0.061657955 | 0.256233836 | 0.037523551 |
| ENSG00000153714.5_2 | LURAP1L | chr9:12775020-12822130 | + | protein_coding | -3.973270591 | 0.063668757 | 4.30813E-06 | 0.000196371 | 0.946458604 | 4.919729195 | 30.24238347 | 29.5003561 | 28.10107994 | 0.655979838 | 1.021484224 | 1.138020609 |
| ENSG00000245532.7_3 | NEAT1 | chr11:65190245-65213011 | + | lincRNA | -3.888630396 | 0.06751583 | 2.17002E-06 | 0.000164057 | 1.962464653 | 5.85109505 | 56.16633394 | 59.80073286 | 54.33707459 | 3.294991058 | 2.947368675 | 2.491501427 |
| ENSG00000169903.6_3 | TM4SF4 | chr3:149191761-149221068 | + | protein_coding | -3.767840094 | 0.07341201 | 1.10388E-05 | 0.000268497 | 0.674601563 | 4.442441657 | 21.9290704 | 21.99518424 | 18.49401234 | 0.857619014 | 0.420093273 | 0.541531484 |
| ENSG00000121895.7_3 | TMEM156 | chr4:38968365-39034542 | - | protein_coding | -3.69659059 | 0.077128583 | 4.56442E-05 | 0.000485032 | 1.403121422 | 5.099712012 | 36.20502023 | 37.84736761 | 26.89560048 | 1.750373298 | 1.241530756 | 2.000603154 |
| ENSG00000241119.1_3 | UGT1A9 | chr2:234580499-234681946 | + | protein_coding | -3.66681695 | 0.078736863 | 5.0978E-06 | 0.000205796 | 0.226840104 | 3.893657054 | 16.110634 | 12.973524 | 12.732533 | 0.2037 | 0.099022 | 0.211525 |
| ENSG00000120738.7_2 | EGR1 | chr5:137801179-137805004 | + | protein_coding | -3.571933308 | 0.084089338 | 2.87984E-06 | 0.000184649 | 0.456062481 | 4.027995789 | 14.471206 | 14.989401 | 16.550371 | 0.339958 | 0.518533 | 0.268674 |
| ENSG00000178607.15_2 | ERN1 | chr17:62116502-62208179 | - | protein_coding | -3.548890021 | 0.085443229 | 6.35184E-07 | 0.000122416 | 1.054906715 | 4.603796735 | 23.22719683 | 23.32878765 | 23.39040985 | 1.230759273 | 0.91505192 | 1.099144964 |
| ENSG00000166750.9_2 | SLFN5 | chr17:33570055-33600674 | + | protein_coding | -3.52502966 | 0.086868103 | 1.662E-07 | 0.000102579 | 0.342842677 | 3.867872336 | 13.3963375 | 14.46253187 | 12.97990275 | 0.292761191 | 0.261799654 | 0.250573088 |
| ENSG00000124882.3_2 | EREG | chr4:75230860-75254468 | + | protein_coding | -3.519143656 | 0.087223237 | 1.52407E-05 | 0.000301175 | 2.824661593 | 6.343805249 | 82.26567347 | 90.41168846 | 69.3978296 | 5.825290554 | 6.978377333 | 5.529619657 |
| ENSG00000146038.11_2 | DCDC2 | chr6:24171984-24358280 | - | protein_coding | -3.44212128 | 0.092006444 | 3.64668E-07 | 0.000113454 | 0.063171999 | 3.505293279 | 10.88715817 | 10.38603737 | 9.817941173 | 0.043150641 | 0.093208426 | 0 |

**Table S2 Top 20 significantly upregulated genes in A549/DDP cells compared to A549 cells** Gene expression was compared between groups using two-tailed Student's t-test. Genes were ranked by P-value, and the top 20 most significant upregulated genes (P < 0.05, log2FC > 0) are presented. (n = 3/group).

| **Metabolite.name** | **KEGGID** | **HMDBID** | **Class** | **A-1** | **A-2** | **A-3** | **B-1** | **B-2** | **B-3** | **C-1** | **C-2** | **C-3** | **D-1** | **D-2** | **D-3** | **P.value** | **VIP** | **PLSDA.VIP** | **RF.MDA** | **Sig** |
| --- | --- | --- | --- | --- | --- | --- | --- | --- | --- | --- | --- | --- | --- | --- | --- | --- | --- | --- | --- | --- |
| Citrate | C00158 | HMDB0000094 | Organic acids and derivatives | 243938033.6 | 246305210.1 | 241886964.2 | 217199925.2 | 216557497.8 | 188224424.9 | 220829160.1 | 182332360.3 | 225128778.4 | 160946245.2 | 174031250.7 | 151939215.7 | 0.001478241 | 1.626695684 | 1.392735947 | 0.020714286 | Sig |
| 6-phosphogluconate | C00345 | HMDB0001316 | Organic oxygen compounds | 6061023.334 | 6871168.017 | 6458505.511 | 5288513.392 | 4869629.948 | 3339407.328 | 4508013.23 | 3920027.026 | 4529985.908 | 3765449.863 | 3960581.099 | 3097852.232 | 0.002737439 | 1.58407553 | 1.285988631 | 0.00975 | Sig |
| Cis-Aconitate | C00417 | HMDB0000072 | Organic acids and derivatives | 2257843.922 | 3213263.394 | 2717159.263 | 1409150.484 | 1005112.334 | 1098876.659 | 2244584.9 | 1325409.412 | 1932793.313 | 897781.1875 | 678265.9972 | 532721.2777 | 0.000690005 | 1.417247833 | 1.103173841 | 0.009 | Sig |
| Succinate | C00042 | HMDB0000254 | Organic acids and derivatives | 1963756.627 | 3345348.915 | 2513458.053 | 1524268.413 | 1022384.283 | 767895.4131 | 1656421.397 | 1272082.621 | 1755607.89 | 867913.7999 | 788725.6872 | 616302.1364 | 0.003413305 | 1.396110757 | 1.099941911 | 0.009833333 | Sig |
| Glutamate | C00025 | HMDB0000148 | Amino acid | 69812120.06 | 84350555.44 | 79752586.67 | 67358185.9 | 53064317.75 | 65204572.94 | 73213188.69 | 64955142.08 | 63333024.65 | 56842912.21 | 58449903.94 | 44983507.02 | 0.015848935 | 1.379660773 | 1.109279813 | 0.004416667 | Sig |
| Phosphoenolpyruvate | C00074 | HMDB0000263 | Organic acids and derivatives | 31903033.07 | 23997427.64 | 21306418.41 | 43653855.8 | 43872324.65 | 59887826 | 45707605.49 | 41003600.32 | 40003003.44 | 54370893.41 | 50980392.95 | 50627185.91 | 0.002062293 | 1.365737175 | 1.099144417 | 0.006214286 | Sig |
| NAD | C00003 | HMDB0001487 | Nucleosides, nucleotides, and analogues | 5174511.336 | 7151170.654 | 7804312.538 | 1231482.061 | 871617.6456 | 503529.5421 | 3601529.773 | 2268995.346 | 2922082.987 | 930999.348 | 1035299.435 | 315653.5963 | 5.61356E-05 | 1.356695865 | 1.064318669 | 0.022333333 | Sig |
| NADH | C00004 | HMDB0001487 | Nucleosides, nucleotides, and analogues | 95352.60335 | 82610.86831 | 145765.5041 | 32554.09387 | 6318.908474 | 14892.02802 | 27931.23831 | 41242.5494 | 42163.5438 | 26512.63472 | 27558.06942 | 907.29367 | 0.001584843 | 1.327533227 | 1.058675969 | 0.014 | Sig |
| D-Glucose 1-phosphate | C00103 | HMDB0001586 | Organic oxygen compounds | 19234847.51 | 23243577.81 | 25725179.79 | 14054080.48 | 14642518.01 | 12479800.32 | 18319118.36 | 16763715.07 | 21554754.91 | 11292488.98 | 13042733.9 | 11362337.55 | 0.00109051 | 1.253493434 | 1.017610988 | -0.00025 | Sig |
| D-Glucose 6-phosphate | C00092 | HMDB0001401 | Organic oxygen compounds | 20227574.19 | 20030058.4 | 23381291.55 | 13541090.88 | 14289084.38 | 12346506.58 | 16747404.32 | 13726182.9 | 20324272.53 | 12093414.83 | 14173018.49 | 11768320.6 | 0.003547603 | 1.239443476 | 1.005715564 | 0.00225 | Sig |
| Fumarate | C00122 | HMDB0000134 | Organic acids and derivatives | 465923.3685 | 776012.8123 | 534699.8701 | 600841.9507 | 361799.879 | 451999.8389 | 669511.0293 | 412428.841 | 569819.2694 | 301709.2214 | 223079.8627 | 252553.2655 | 0.040946697 | 1.203797963 | 0.964384168 | 0.009214286 | Sig |
| NADP | C00006 | HMDB0000221 | Nucleosides, nucleotides, and analogues | 309684.2356 | 368059.0956 | 421288.3002 | 210537.777 | 139934.2601 | 116809.8078 | 317312.0661 | 287151.9879 | 238931.9498 | 161106.8194 | 185765.8514 | 56697.34701 | 0.002380506 | 1.167429296 | 0.972235028 | 0.003916667 | Sig |
| D-Fructose 6-phosphate | C00085 | HMDB0000124 | Organic oxygen compounds | 17668661.29 | 20850021.73 | 24090828.03 | 14756859.83 | 13392758.27 | 12184594.82 | 18277336.39 | 14625498.8 | 20757736.97 | 13260306.54 | 13703031.12 | 10453840.72 | 0.010329367 | 1.112444468 | 0.941341446 | 0.000166667 | Sig |
| L-Malate | C00149 | HMDB0000156 | Organic acids and derivatives | 114300217.4 | 136706448.6 | 117546884.6 | 134419168.5 | 105049537.5 | 126849765.3 | 137147959.4 | 94788599.38 | 113241157.4 | 83253781.23 | 100919883.6 | 69699326.54 | 0.064283426 | 1.251466043 | 1.096399384 | 0.002666667 | NoSig |
| Isocitrate | C00311 | HMDB0001874 | Organic acids and derivatives | 882195.7285 | 873763.5712 | 653827.5514 | 606500.3906 | 551898.4368 | 944924.0937 | 804467.2101 | 636162.6316 | 862908.1721 | 511842.7948 | 528792.3567 | 472257.0122 | 0.109847474 | 1.093269161 | 1.10148572 | 0.019833333 | NoSig |
| Succinyl-CoA | C00091 | HMDB0001022 | Lipids and lipid-like molecules | 9558.481432 | 2709.61589 | 5803.547554 | 6986.382529 | 11355.81813 | 13014.22616 | 6225.925656 | 5955.887933 | 6958.994626 | 9638.740098 | 18866.33818 | 13836.68878 | 0.050104916 | 1.011555659 | 0.86084335 | -0.000833333 | NoSig |
| 2-phosphoglycerate | C00631 | HMDB0003391 | Organic oxygen compounds | 55576862.65 | 53011090.42 | 53705486.75 | 50158159.92 | 49930701.18 | 64463093.27 | 78080578 | 63292372 | 83593215.77 | 58199648.07 | 70734269.51 | 60379550.36 | 0.030417306 | 0.972489939 | 1.359628983 | 0.005535714 | NoSig |
| L-Lactate | C00186 | HMDB0001311 | Organic acids and derivatives | 69549657.69 | 95519303.12 | 85145453.92 | 75562122.57 | 62178877.95 | 60988400.85 | 85850638.31 | 65763833.74 | 72810133.45 | 68699174.89 | 66082590.58 | 58089610.74 | 0.134854579 | 0.954083117 | 1.006292273 | -0.0015 | NoSig |
| D-Ribose 5-phosphate | C00117 | HMDB0001548 | Organic oxygen compounds | 20004911.54 | 18567160.15 | 21020394.95 | 18849742.34 | 9696943.703 | 21941732.8 | 25782857.66 | 20999622.19 | 16831078.07 | 10769305.98 | 13145751.53 | 8494293.813 | 0.058130305 | 0.94114519 | 0.844522617 | -0.0025 | NoSig |
| NADPH | C00005 | HMDB0000221 | Nucleosides, nucleotides, and analogues | 35125.29836 | 73460.24416 | 57012.89818 | 55685.09805 | 53761.79326 | 40304.75605 | 69699.34839 | 33164.70114 | 43703.89732 | 42349.52633 | 37432.27468 | 27722.42201 | 0.458547829 | 0.900672197 | 0.816643801 | 0.001428571 | NoSig |
| 3-phosphoglycerate | C00597 | HMDB0060180 | Organic oxygen compounds | 64913105.37 | 59099191.71 | 61218757.1 | 53736394.41 | 56504377.84 | 74279992.32 | 88317943.27 | 75775915.63 | 91880419.13 | 62905751.58 | 79203914.35 | 67287361.19 | 0.024774533 | 0.871321036 | 1.265353324 | 0.00975 | NoSig |
| a-Ketoglutaric acid | C00026 | HMDB0000208 | Organic acids and derivatives | 17904390.94 | 16145212.2 | 16608047.41 | 16799236.57 | 17369160.89 | 19046838.29 | 12909466.65 | 14897510.46 | 15195461.54 | 16505284.81 | 16350866.39 | 14912366.07 | 0.022241852 | 0.865829262 | 1.587474791 | 0.013083333 | NoSig |
| ATP | C00002 | HMDB0000538 | Nucleosides, nucleotides, and analogues | 11152104.73 | 12031283.84 | 9924320.315 | 6317194.487 | 4222869.49 | 2034475.775 | 13869259.73 | 10905493.18 | 8641417.371 | 3923613.805 | 6558093.102 | 2870865.876 | 0.002990851 | 0.731927678 | 0.881081577 | 0.006166667 | NoSig |
| ADP | C00008 | HMDB0001341 | Nucleosides, nucleotides, and analogues | 10286940.55 | 12202924.84 | 10172550.65 | 8562341.557 | 6651577.765 | 4120719.157 | 10924910.41 | 8451661.58 | 10482426.82 | 6964867.751 | 9355411.648 | 5012174.066 | 0.042254467 | 0.697572937 | 0.947402467 | 0 | NoSig |
| Thiamine pyrophosphate | C00068 | HMDB0062636 | Organoheterocyclic compounds | 152251.3994 | 147847.8634 | 142719.2897 | 89902.57564 | 55676.65406 | 92918.68323 | 118504.12 | 122787.0314 | 163993.7581 | 116795.6788 | 101370.7372 | 61235.74725 | 0.014074483 | 0.687717753 | 0.83675863 | 0.009785714 | NoSig |
| UDPglucose | C00029 | HMDB0000286 | Nucleosides, nucleotides, and analogues | 33270326.3 | 36556654.05 | 35363863 | 25391874.43 | 20491898.18 | 23163837.85 | 34543024.38 | 32047063.62 | 37631275.15 | 25285967.19 | 28962420.14 | 19336253.02 | 0.002212552 | 0.682862045 | 0.930925316 | 0.0105 | NoSig |
| GTP | C00044 | HMDB0003379 | Homogeneous non-metal compounds | 605156.2163 | 466719.4725 | 330901.7434 | 215040.8306 | 185430.7402 | 177213.9081 | 534067.2705 | 485181.7304 | 309688.9036 | 180912.1192 | 353434.6467 | 146750.2876 | 0.025320931 | 0.676683096 | 0.708324034 | -0.000166667 | NoSig |
| GDP | C00035 | HMDB0001201 | Nucleosides, nucleotides, and analogues | 1808696.486 | 1900059.952 | 1541061.929 | 1485834.621 | 1161614.96 | 1109000.448 | 1908319.162 | 1671530.084 | 1520634.781 | 1164347.886 | 1707836.08 | 932000.3896 | 0.084460064 | 0.671361892 | 0.814118567 | 0 | NoSig |
| ADPG | C00498 | HMDB0006557 | Nucleosides, nucleotides, and analogues | 307109.548 | 229585.7646 | 288392.3423 | 200690.0288 | 162673.4637 | 149701.4131 | 317806.8904 | 262532.9948 | 261368.6477 | 160636.2615 | 215088.0875 | 171355.5986 | 0.004709631 | 0.639803587 | 0.826090632 | 0.000916667 | NoSig |
| D-Fructose 1,6-bisphosphate | C00354 | HMDB0001058 | Organic oxygen compounds | 59915133.53 | 68580627.5 | 71267484.39 | 39031950.46 | 31384656.78 | 45876997.52 | 82484880.02 | 58929139.03 | 75834053.86 | 40179297.16 | 42999740.65 | 32465420.85 | 0.001304784 | 0.639008894 | 0.884873724 | 0.0085 | NoSig |
| AMP | C00020 | HMDB0014839 | Benzenoids | 6660219.541 | 10252390.91 | 10350105.59 | 8197840.156 | 5736744.803 | 3923316.114 | 9644343.441 | 6793210.418 | 10735924.02 | 6216725.081 | 6575868.445 | 6477835.098 | 0.128458181 | 0.495018418 | 0.967829269 | 0.00125 | NoSig |
| Trans-Aconitate | C02341 | HMDB0000958 | Organic acids and derivatives | 115596.0153 | 105692.8539 | 89925.72626 | 87763.32839 | 64058.9053 | 78768.53215 | 127491.885 | 99769.09435 | 183418.9517 | 115965.2313 | 107715.9071 | 90006.50044 | 0.086703228 | 0.468607269 | 1.202356275 | 0.004583333 | NoSig |
| Glyceraldehyde 3-phosphate | C00661 | HMDB0001112 | Organic oxygen compounds | 26141534.36 | 17608992.04 | 27522731.42 | 47569584.5 | 21961948.59 | 55859851.06 | 57226268.67 | 48623388.7 | 32860738.99 | 31277538.39 | 34070133.43 | 24472559.01 | 0.133584117 | 0.372769657 | 0.59310961 | 0.0025 | NoSig |
| Ribulose-1, 5 bisphosphate | C01182 | HMDB0304322 | Organic oxygen compounds | 1072966.843 | 1039479.052 | 1038641.538 | 1263827.282 | 911097.2979 | 1330542.764 | 1393172.251 | 1229282.617 | 1169680.062 | 899498.9753 | 1037862.647 | 898926.4484 | 0.080841579 | 0.288592514 | 0.617674086 | 0.016511905 | NoSig |
| cAMP | C00575 | HMDB0000058 | Nucleosides, nucleotides, and analogues | 6649.464679 | 12185.76329 | 12442.19645 | 9719.409326 | 13833.79144 | 7910.837526 | 6398.102753 | 21408.79269 | 2987.677771 | 15182.21763 | 6062.377624 | 4989.5504 | 0.981334034 | 0.217121207 | 0.499731002 | -0.004166667 | NoSig |
| Acetyl-CoA | C00024 | HMDB0001206 | Organic oxygen compounds | 138099.2022 | 81049.70833 | 73678.09926 | 149855.035 | 83543.07419 | 135396.3384 | 182729.4005 | 119379.3313 | 147837.8076 | 71604.57379 | 164394.79 | 67662.61005 | 0.411052796 | 0.214809035 | 0.655548983 | -0.004166667 | NoSig |
| Dihydroxyacetone phosphate | C00111 | HMDB0001473 | Organic oxygen compounds | 23955176.3 | 18081200.97 | 22784678.91 | 38259408.48 | 19420961.33 | 44828573.7 | 42970253.09 | 39738300.32 | 29085786.3 | 27602871.25 | 27768400.13 | 15738787.89 | 0.133316728 | 0.208854223 | 0.542574078 | 0.002833333 | NoSig |

**Table S3 Effects of SJZD combination medication on TCA cycle metabolites and glycolytic pathway in glutamine-deprived A549/DDP cells.** A: control group (NS-medicated serum); B: CDDP monotherapy group; C: SJZD group (SJZD-medicated serum); D: SJZD-CDDP combination group (n = 3/group). The data represent the mean ± SD.

| **Metabolite.name** | **KEGGID** | **HMDBID** | **Class** | **D-1** | **D-2** | **D-3** | **B-1** | **B-2** | **B-3** | **FC** | **P.value** | **VIP** | **PLSDA_VIP** | **Log2FC** | **NLP** | **Sig** |
| --- | --- | --- | --- | --- | --- | --- | --- | --- | --- | --- | --- | --- | --- | --- | --- | --- |
| Fumarate | C00122 | HMDB0000134 | Organic acids and derivatives | 301709.2214 | 223079.8627 | 252553.2655 | 600841.9507 | 361799.879 | 451999.8389 | 0.549497705 | 0.044324672 | 1.637141371 | 1.518987023 | -0.86381464 | 1.353354469 | **Sig** |
| Cis-Aconitate | C00417 | HMDB0000072 | Organic acids and derivatives | 897781.1875 | 678265.9972 | 532721.2777 | 1409150.484 | 1005112.334 | 1098876.659 | 0.600251848 | 0.044380695 | 1.63130155 | 1.522978749 | -0.736360156 | 1.352805906 | **Sig** |
| Citrate | C00158 | HMDB0000094 | Organic acids and derivatives | 160946245.2 | 174031250.7 | 151939215.7 | 217199925.2 | 216557497.8 | 188224424.9 | 0.782847141 | 0.017353521 | 1.767187159 | 1.644640095 | -0.35319746 | 1.760612397 | Non-Sig |
| L-Malate | C00149 | HMDB0000156 | Organic acids and derivatives | 83253781.23 | 100919883.6 | 69699326.54 | 134419168.5 | 105049537.5 | 126849765.3 | 0.693039012 | 0.04111967 | 1.641675577 | 1.528217073 | -0.52899153 | 1.385950384 | Non-Sig |
| Trans-Aconitate | C02341 | HMDB0000958 | Organic acids and derivatives | 115965.2313 | 107715.9071 | 90006.50044 | 87763.32839 | 64058.9053 | 78768.53215 | 1.360365137 | 0.054905807 | 1.593591699 | 1.594461544 | 0.443993939 | 1.260381724 | Non-Sig |
| NADPH | C00005 | HMDB0000221 | Nucleosides, nucleotides, and analogues | 42349.52633 | 37432.27468 | 27722.42201 | 55685.09805 | 53761.79326 | 40304.75605 | 0.717883408 | 0.095147731 | 1.459503734 | 1.360250392 | -0.47817854 | 1.021601563 | Non-Sig |
| a-Ketoglutaric acid | C00026 | HMDB0000208 | Organic acids and derivatives | 16505284.81 | 16350866.39 | 14912366.07 | 16799236.57 | 17369160.89 | 19046838.29 | 0.897647386 | 0.097851288 | 1.43508323 | 1.360904698 | -0.155779259 | 1.009433452 | Non-Sig |
| D-Glucose 1-phosphate | C00103 | HMDB0001586 | Organic oxygen compounds | 11292488.98 | 13042733.9 | 11362337.55 | 14054080.48 | 14642518.01 | 12479800.32 | 0.866942264 | 0.101669126 | 1.443716347 | 1.362192117 | -0.205992177 | 0.992810908 | Non-Sig |
| Ribulose-1, 5 bisphosphate | C01182 | HMDB0304322 | Organic oxygen compounds | 899498.9753 | 1037862.647 | 898926.4484 | 1263827.282 | 911097.2979 | 1330542.764 | 0.80910412 | 0.181550319 | 1.243552348 | 1.15638697 | -0.305602726 | 0.741002984 | Non-Sig |
| Isocitrate | C00311 | HMDB0001874 | Organic acids and derivatives | 511842.7948 | 528792.3567 | 472257.0122 | 606500.3906 | 551898.4368 | 944924.0937 | 0.719286681 | 0.1878299 | 1.219919156 | 1.167818189 | -0.475361204 | 0.726235273 | Non-Sig |
| D-Ribose 5-phosphate | C00117 | HMDB0001548 | Organic oxygen compounds | 10769305.98 | 13145751.53 | 8494293.813 | 18849742.34 | 9696943.703 | 21941732.8 | 0.641916544 | 0.198460742 | 1.203957124 | 1.124246553 | -0.639542352 | 0.702325389 | Non-Sig |
| Succinate | C00042 | HMDB0000254 | Organic acids and derivatives | 867913.7999 | 788725.6872 | 616302.1364 | 1524268.413 | 1022384.283 | 767895.4131 | 0.685747061 | 0.212490177 | 1.189687874 | 1.136816884 | -0.544251562 | 0.672661142 | Non-Sig |
| 6-phosphogluconate | C00345 | HMDB0001316 | Organic oxygen compounds | 3765449.863 | 3960581.099 | 3097852.232 | 5288513.392 | 4869629.948 | 3339407.328 | 0.801914618 | 0.240657094 | 1.130796653 | 1.064943443 | -0.318479457 | 0.618601331 | Non-Sig |
| Glutamate | C00025 | HMDB0000148 | Amino acid | 56842912.21 | 58449903.94 | 44983507.02 | 67358185.9 | 53064317.75 | 65204572.94 | 0.863431812 | 0.241430305 | 1.11562663 | 1.083791397 | -0.211845846 | 0.617208217 | Non-Sig |
| 2-phosphoglycerate | C00631 | HMDB0003391 | Organic oxygen compounds | 58199648.07 | 70734269.51 | 60379550.36 | 50158159.92 | 49930701.18 | 64463093.27 | 1.150478392 | 0.251893633 | 1.113406293 | 1.032841514 | 0.202233888 | 0.598782809 | Non-Sig |
| Dihydroxyacetone phosphate | C00111 | HMDB0001473 | Organic oxygen compounds | 27602871.25 | 27768400.13 | 15738787.89 | 38259408.48 | 19420961.33 | 44828573.7 | 0.693696148 | 0.290148942 | 1.022500805 | 0.975032892 | -0.527624221 | 0.537379009 | Non-Sig |
| Succinyl-CoA | C00091 | HMDB0001022 | Lipids and lipid-like molecules | 9638.740098 | 18866.33818 | 13836.68878 | 6986.382529 | 11355.81813 | 13014.22616 | 1.350337757 | 0.31851769 | 0.988251382 | 0.965660614 | 0.43332031 | 0.496866443 | Non-Sig |
| Glyceraldehyde 3-phosphate | C00661 | HMDB0001112 | Organic oxygen compounds | 31277538.39 | 34070133.43 | 24472559.01 | 47569584.5 | 21961948.59 | 55859851.06 | 0.716318999 | 0.325667994 | 0.962773607 | 0.909655709 | -0.481325887 | 0.487224921 | Non-Sig |
| 3-phosphoglycerate | C00597 | HMDB0060180 | Organic oxygen compounds | 62905751.58 | 79203914.35 | 67287361.19 | 53736394.41 | 56504377.84 | 74279992.32 | 1.134815519 | 0.362295105 | 0.91804966 | 0.857134767 | 0.182457785 | 0.440937533 | Non-Sig |
| D-Fructose 6-phosphate | C00085 | HMDB0000124 | Organic oxygen compounds | 13260306.54 | 13703031.12 | 10453840.72 | 14756859.83 | 13392758.27 | 12184594.82 | 0.927678407 | 0.483275998 | 0.71090952 | 0.801767979 | -0.108303334 | 0.315804774 | Non-Sig |
| D-Glucose 6-phosphate | C00092 | HMDB0001401 | Organic oxygen compounds | 12093414.83 | 14173018.49 | 11768320.6 | 13541090.88 | 14289084.38 | 12346506.58 | 0.946687287 | 0.490697225 | 0.701045892 | 0.726043484 | -0.079040147 | 0.309186398 | Non-Sig |
| Thiamine pyrophosphate | C00068 | HMDB0062636 | Organoheterocyclic compounds | 116795.6788 | 101370.7372 | 61235.74725 | 89902.57564 | 55676.65406 | 92918.68323 | 1.171507791 | 0.540802912 | 0.640501206 | 0.834623585 | 0.228366549 | 0.266960978 | Non-Sig |
| Acetyl-CoA | C00024 | HMDB0001206 | Organic oxygen compounds | 71604.57379 | 164394.79 | 67662.61005 | 149855.035 | 83543.07419 | 135396.3384 | 0.82339085 | 0.593413392 | 0.54900075 | 0.600534599 | -0.280350678 | 0.226642657 | Non-Sig |
| GTP | C00044 | HMDB0003379 | Homogeneous non-metal compounds | 180912.1192 | 353434.6467 | 146750.2876 | 215040.8306 | 185430.7402 | 177213.9081 | 1.179010168 | 0.623910972 | 0.513520295 | 0.64253732 | 0.23757616 | 0.204877377 | Non-Sig |
| Phosphoenolpyruvate | C00074 | HMDB0000263 | Organic acids and derivatives | 54370893.41 | 50980392.95 | 50627185.91 | 43653855.8 | 43872324.65 | 59887826 | 1.058098047 | 0.631486054 | 0.513827199 | 0.479021119 | 0.081473318 | 0.199636236 | Non-Sig |
| ADPG | C00498 | HMDB0006557 | Nucleosides, nucleotides, and analogues | 160636.2615 | 215088.0875 | 171355.5986 | 200690.0288 | 162673.4637 | 149701.4131 | 1.066297737 | 0.642449775 | 0.472256794 | 0.642585248 | 0.09261033 | 0.192160819 | Non-Sig |
| UDPglucose | C00029 | HMDB0000286 | Nucleosides, nucleotides, and analogues | 25285967.19 | 28962420.14 | 19336253.02 | 25391874.43 | 20491898.18 | 23163837.85 | 1.065708717 | 0.655421465 | 0.472293657 | 0.72629747 | 0.09181317 | 0.18347934 | Non-Sig |
| cAMP | C00575 | HMDB0000058 | Nucleosides, nucleotides, and analogues | 15182.21763 | 6062.377624 | 4989.5504 | 9719.409326 | 13833.79144 | 7910.837526 | 0.833781901 | 0.660224482 | 0.447927396 | 0.475412027 | -0.262258039 | 0.180308375 | Non-Sig |
| NADP | C00006 | HMDB0000221 | Nucleosides, nucleotides, and analogues | 161106.8194 | 185765.8514 | 56697.34701 | 210537.777 | 139934.2601 | 116809.8078 | 0.863654392 | 0.684533986 | 0.420361851 | 0.668705106 | -0.211473989 | 0.164604985 | Non-Sig |
| AMP | C00020 | HMDB0014839 | Benzenoids | 6216725.081 | 6575868.445 | 6477835.098 | 8197840.156 | 5736744.803 | 3923316.114 | 1.079098184 | 0.724148579 | 0.349970637 | 0.575594988 | 0.109826138 | 0.140172317 | Non-Sig |
| ADP | C00008 | HMDB0001341 | Nucleosides, nucleotides, and analogues | 6964867.751 | 9355411.648 | 5012174.066 | 8562341.557 | 6651577.765 | 4120719.157 | 1.103328282 | 0.729847659 | 0.35729381 | 0.644268854 | 0.141862112 | 0.136767781 | Non-Sig |
| NAD | C00003 | HMDB0001487 | Nucleosides, nucleotides, and analogues | 930999.348 | 1035299.435 | 315653.5963 | 1231482.061 | 871617.6456 | 503529.5421 | 0.875441868 | 0.742655146 | 0.343607987 | 0.618092707 | -0.191916712 | 0.129212805 | Non-Sig |
| L-Lactate | C00186 | HMDB0001311 | Organic acids and derivatives | 68699174.89 | 66082590.58 | 58089610.74 | 75562122.57 | 62178877.95 | 60988400.85 | 0.970522605 | 0.747386984 | 0.341684048 | 0.693635969 | -0.04316628 | 0.12645447 | Non-Sig |
| ATP | C00002 | HMDB0000538 | Nucleosides, nucleotides, and analogues | 3923613.805 | 6558093.102 | 2870865.876 | 6317194.487 | 4222869.49 | 2034475.775 | 1.061873679 | 0.882903774 | 0.148522306 | 0.532457296 | 0.086612152 | 0.054086627 | Non-Sig |
| GDP | C00035 | HMDB0001201 | Nucleosides, nucleotides, and analogues | 1164347.886 | 1707836.08 | 932000.3896 | 1485834.621 | 1161614.96 | 1109000.448 | 1.012707297 | 0.953840553 | 0.062645823 | 0.50535062 | 0.018217252 | 0.020524217 | Non-Sig |
| D-Fructose 1,6-bisphosphate | C00354 | HMDB0001058 | Organic oxygen compounds | 40179297.16 | 42999740.65 | 32465420.85 | 39031950.46 | 31384656.78 | 45876997.52 | 0.994418041 | 0.96902643 | 0.026108023 | 0.292620271 | -0.008075624 | 0.013664378 | Non-Sig |
| NADH | C00004 | HMDB0001487 | Nucleosides, nucleotides, and analogues | 26512.63472 | 27558.06942 | 907.29367 | 32554.09387 | 6318.908474 | 14892.02802 | 1.022560527 | 0.973965025 | 0.038038894 | 0.661341154 | 0.032186242 | 0.011456638 | Non-Sig |

**Table S4 Effects of SJZD combination medication on TCA cycle metabolites and glycolytic pathway in glutamine-deprived A549/DDP cells.** B: CDDP monotherapy group; D: SJZD-CDDP combination group (n = 3/group). The data represent the mean ± SD.
